# B-Cell Immunophenotyping to Predict Vaccination Outcome in the Immunocompromised - A Systematic Review

**DOI:** 10.3389/fimmu.2021.690328

**Published:** 2021-09-07

**Authors:** Annieck M. Diks, Lisanne A. Overduin, Laurens D. van Leenen, Lennert Slobbe, Hetty Jolink, Leonardus G. Visser, Jacques J. M. van Dongen, Magdalena A. Berkowska

**Affiliations:** ^1^Department of Immunology, Leiden University Medical Center (LUMC), Leiden, Netherlands; ^2^Department of Infectious Diseases, Leiden University Medical Center (LUMC), Leiden, Netherlands; ^3^Department of Internal Medicine, Section of Infectious Diseases, Institute for Tropical Diseases, Erasmus Medical Center (MC), Rotterdam, Netherlands

**Keywords:** B cells, vaccination, extremities of life, immunodeficiency, immunosuppression, immune monitoring

## Abstract

Vaccination is the most effective measure to prevent infections in the general population. Its efficiency strongly depends on the function and composition of the immune system. If the immune system lacks critical components, patients will not be fully protected despite a completed vaccination schedule. Antigen-specific serum immunoglobulin levels are broadly used correlates of protection. These are the products of terminally differentiated B cells – plasma cells. Here we reviewed the literature on how aberrancies in B-cell composition and function influence immune responses to vaccinations. In a search through five major literature databases, 6,537 unique articles published from 2000 and onwards were identified. 75 articles were included along three major research lines: extremities of life, immunodeficiency and immunosuppression. Details of the protocol can be found in the International Prospective Register of Systematic Reviews [PROSPERO (registration number CRD42021226683)]. The majority of articles investigated immune responses in adults, in which vaccinations against pneumococci and influenza were strongly represented. Lack of baseline information was the most common reason of exclusion. Irrespective of study group, three parameters measured at baseline seemed to have a predictive value in assessing vaccine efficacy: (1) distribution of B-cell subsets (mostly a reduction in memory B cells), (2) presence of exhausted/activated B cells, or B cells with an aberrant phenotype, and (3) pre-existing immunological memory. In this review we showed how pre-immunization (baseline) knowledge of circulating B cells can be used to predict vaccination efficacy. We hope that this overview will contribute to optimizing vaccination strategies, especially in immunocompromised patients.

## Introduction

Vaccination is the most effective measure to prevent infectious diseases in the general population (1). However, its efficiency strongly depends on the fitness of the immune system. If the immune system is suppressed or lacks critical components, individuals are not fully protected despite a completed vaccination schedule. As a result, the immunocompromised population is at increased risk of morbidity and mortality caused by vaccine-preventable diseases (2–4). Aside from people suffering from inherited or acquired immunodeficiencies, also relatively healthy individuals can have weakened immune responses, i.e. early in life or as a consequence of aging (5–8). In this review, we evaluated the current literature on vaccine responsiveness in individuals with (temporarily) altered B-cell systems.

An effective immune response depends on the cooperation of multiple cell types. During the immune response, innate immune cells are rapidly recruited to the place of damage or infection (9–11). They initiate the immune response by means of local inflammation, but also act as antigen (Ag)-presenting cells (10, 11). The innate response is followed by activation of adaptive immune cells (T and B cells), which results in the formation of effector and memory cells (8).

**Figure 1:**
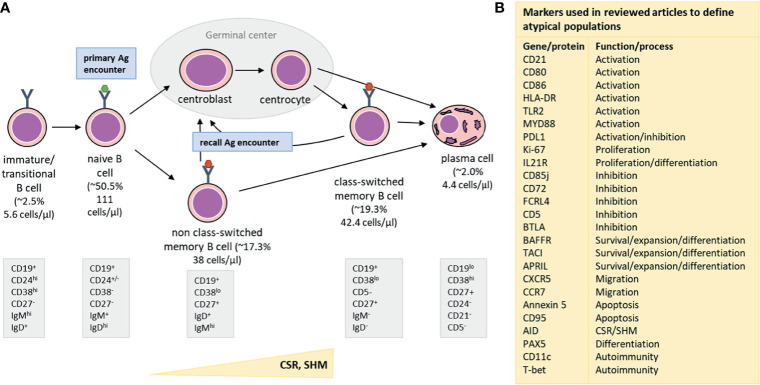
Simplified representation of major B-cell subsets detectable in blood **(A)** along with an overview of markers **(B)** used to define atypical B-cell populations. **(A)** From left to right: Immature/Transitional B cells are recent bone marrow migrants and present in low numbers in the peripheral blood. They mature into naive B cells, which constitute the major part of the circulating B-cell population. The number of naive B cells with unique B-cell receptors forms the naive B-cell repertoire, which is crucial for recognition of neoantigens (primary antigen encounters). Naive B cells which encountered T-cell dependent antigens (e.g. a protein), will enter germinal centers to receive T-cell help. As a consequence, they will upregulate AID (Activation Induced Cytidine Deaminase) and subsequently improve affinity for antigen by introducing Somatic Hypermutations (SHM) and change effector functions in the process of Class-Switch Recombination (CSR). Only B cells that express receptors with increased affinity to the encountered antigen survive and can leave the germinal center as class-switched memory B cells (MBC) or as plasma cells. When a B cell is activated by a T-cell independent antigen (e.g. a polysaccharide, nucleic acid or lipid), it does not enter the germinal center, but differentiates into a non class-switched MBC instead. Class-switched and non class-switched MBCs make up a large part of the circulating B-cell compartment, and are present in other parts of the peripheral lymphoid system, such as the spleen or lymph nodes. These MBCs are important during recall responses (recall antigen encounter) when they can re-enter germinal centers and undergo further processes of affinity maturation and class-switching. Lastly, plasma cells are the terminal effector B cells and responsible for massive antibody production after antigen encounter. Upon infection or vaccination, a transient peak of plasma cell numbers is observed, but in steady state, plasma cell numbers are low. Part of the plasma cells generated during an immune response becomes long-lived plasma cells that migrate to the bone marrow, where they can stay for many years and produce low quantities of antibodies, which are detectable in serum. Underneath each phenotypic description we provide reference values for each of the populations. The median % (of total B cells) and median cell count in the periphery (cells/µl) are indicated (12). These values are based on the publication by Blanco et al., JACI, 2018, who derived these numbers from a cohort of 32 healthy adults, aged 18-39 years. **(B)** An overview of different cellular and genetic markers that were used in the reviewed publications to define atypical B-cell subsets, such as exhausted, tissue-like, anergic, activated or immunesenescent phenotypes. The right column indicates the most prominent function or process involvement for each marker.

In healthy adults, the majority of B cells are of the naive mature or memory B-cell (MBC) phenotype [**Figure 1**, adjusted from (13, 14)]. Additionally, low numbers of transitional/immature B cells (recent bone marrow migrants) and plasma cells (terminally-differentiated effector B cells) can be detected in peripheral blood (12). While the breadth of the naive B-cell repertoire (number of naive B cells carrying a unique B-cell receptor) is crucial in the response to neoantigens, the diversity within the MBC compartment, shaped by previous antigen encounters, plays an important role in recall responses, such as to a booster vaccination. Within MBCs, two major subpopulations can be defined: the non class-switched MBCs and class-switched MBCs. Most non class-switched MBCs are believed to be derived from T-cell independent immune responses to antigens such as polysaccharides, nucleic acids and lipids. In contrast, formation of class-switched MBCs is mostly T-cell dependent and takes place in germinal centers upon recognition of protein antigens (15). However, it should be noted that several exceptions to these general observations have been described, such as T-cell independent IgA responses in mucosa-associated lymphoid tissues or T-cell dependent origin of a part of IgM^+^ MBCs (16). Additionally, comparative studies in B cells are further complicated by the existence of multiple different phenotypic descriptions and the existence of atypical B-cell populations associated with both physiological and pathological processes (**Supplemental Table 1**).

In general, vaccine trials evaluate immune response in healthy adults. However, in several health conditions/situations the immune system is not comparable to that of a healthy adult, which may have an impact on vaccination efficacy and, in case of live-attenuated vaccines, also on safety. For example, in the elderly, B-cell numbers can be lowered and show signs of immunosenescence, the repertoire is restricted, and protective responses mostly rely on immunological memory (17). Other situations in which immune responses may be impaired include, but are not limited to, patients rebuilding their immune system after bone marrow transplantation or B-cell depletion, immunodeficient individuals with impaired cell maturation or human immunodeficiency virus (HIV)-infected individuals showing premature signs of immune system aging (5–7). In such situations, B-cell composition may be altered, and other B-cell phenotypes, such as exhausted or age-associated B cells, may get a more prominent place in the B-cell compartment.

The ability to predict vaccination responses in immuno-compromised individuals is crucial to ensure safety and optimal protection. Here we set out to determine if this goal can be achieved by assessing the B-cell compartment by one of multiple available techniques, e.g. flow cytometry, ELISpot or gene expression analysis. We reviewed whether B-cell characteristics of immuno-compromised patients and aged individuals correlated with vaccination outcomes.

## Methods

### Search Strategy

This review was conducted in accordance with the Preferred Reporting Items for Systematic reviews and Meta-Analyses checklist (PRISMA) (18). The protocol for this review can be found in the International Prospective Register of Systematic Reviews (PROSPERO) under registration number CRD42021226683 (https://www.crd.york.ac.uk/prospero/display_record.php?RecordID=226683).

Literature databases of PubMed, Embase, Web of Science, COCHRANE Library, and Academic Search Premier were searched using the search strategy provided in **Supplemental Text 1**. The search strategy included components for ‘B cells’, ‘vaccination’ and a variety of immunocompromising conditions affecting the B-cell compartment.

### Inclusion and Exclusion Criteria

We included studies that used standard vaccines included in national immunization programs or travel recommendations. Studies on novel or anti-cancer vaccines were excluded. B-cell status at baseline in blood or bone marrow had to be well-documented, either quantitatively or qualitatively. Studies in immunocompromised patients with an unaffected B-cell compartment as well as healthy children, young and middle-aged adults were excluded. Lack of detectable B cells at the time of vaccination was another reason for exclusion. Both humoral and cellular outcomes were eligible for inclusion.

Studies were excluded if no English full text was available. Case reports, review articles, editorials, meeting abstracts, book chapters or conference summaries were excluded as well. They could however be used for ‘snowballing’: finding original research articles not retrieved by the initial literature search. Animal studies were excluded, as were studies published before 2000. Other reasons for exclusion included lack of quantitative or functional B-cell defect (wrong cohort), wrong type of vaccine, wrong timing of vaccination (e.g. before immunosuppressive treatment), insufficient baseline details, insufficient follow-up details, wrong tissue or wrong scope (B-cell data insufficiently discussed).

Specific inclusion and exclusion criteria were added for the three subtopics (extremities of life, immunodeficiencies, and immunosuppression). In the extremities of life, term and preterm infants were maximum 12 months (m) old, and elderly at least 64 years old. For publications on immunodeficiencies and immunosuppression, quantitative data of at least two B-cell subsets or ELISpot data were required. Manuscripts that used neoantigen vaccination to evaluate B-cell defects or immune system reconstitution were included only if more than serological outcomes were reported post-vaccination.

### Primary and Secondary Endpoints

The primary endpoint was vaccine efficacy as measured by qualitative and quantitative changes in B cells, plasma cells and Ag-specific immunoglobulin (Ig) levels, and their kinetics over time following vaccination. A secondary endpoint was the feasibility of the use of neoantigen vaccination to evaluate immunological defects.

### Selection Process

Each article identified by the search strategies in **Supplemental Text 1** was independently screened by two reviewers, first based on title and abstract, and then on full text. Unanimously selected studies were included, and unanimously rejected studies were excluded. Reasons for exclusion were documented. Studies that were assessed eligible by only one of the reviewers were reassessed to reach consensus. In addition, the citations and reference lists from review articles found in the initial search were checked to ensure that no relevant studies had been missing in the initial search.

### Data Collection Process

Data was extracted from the included studies, using a data extraction sheet developed by all reviewers and reviewed by co-authors. The extracted data included article title, authors, country, year of publication, study design, study cohort(s), type and timing of vaccination, methods of measurement of baseline B-cell status, methods of measurement of vaccination response, baseline B-cell status of study cohort(s), B-cell related post-vaccination outcomes of study cohort(s), and correlations between B-cell parameters at baseline and post-vaccination outcomes.

### Risk of Bias Assessment

The risk of bias was assessed by one of the reviewers using the ROBINS-I tool for non-randomized studies and the RoB 2 tool for randomized studies (19, 20). This assessment was reviewed by the other reviewers.

## Results

### Characteristics of Included Studies

**Figure 2:**
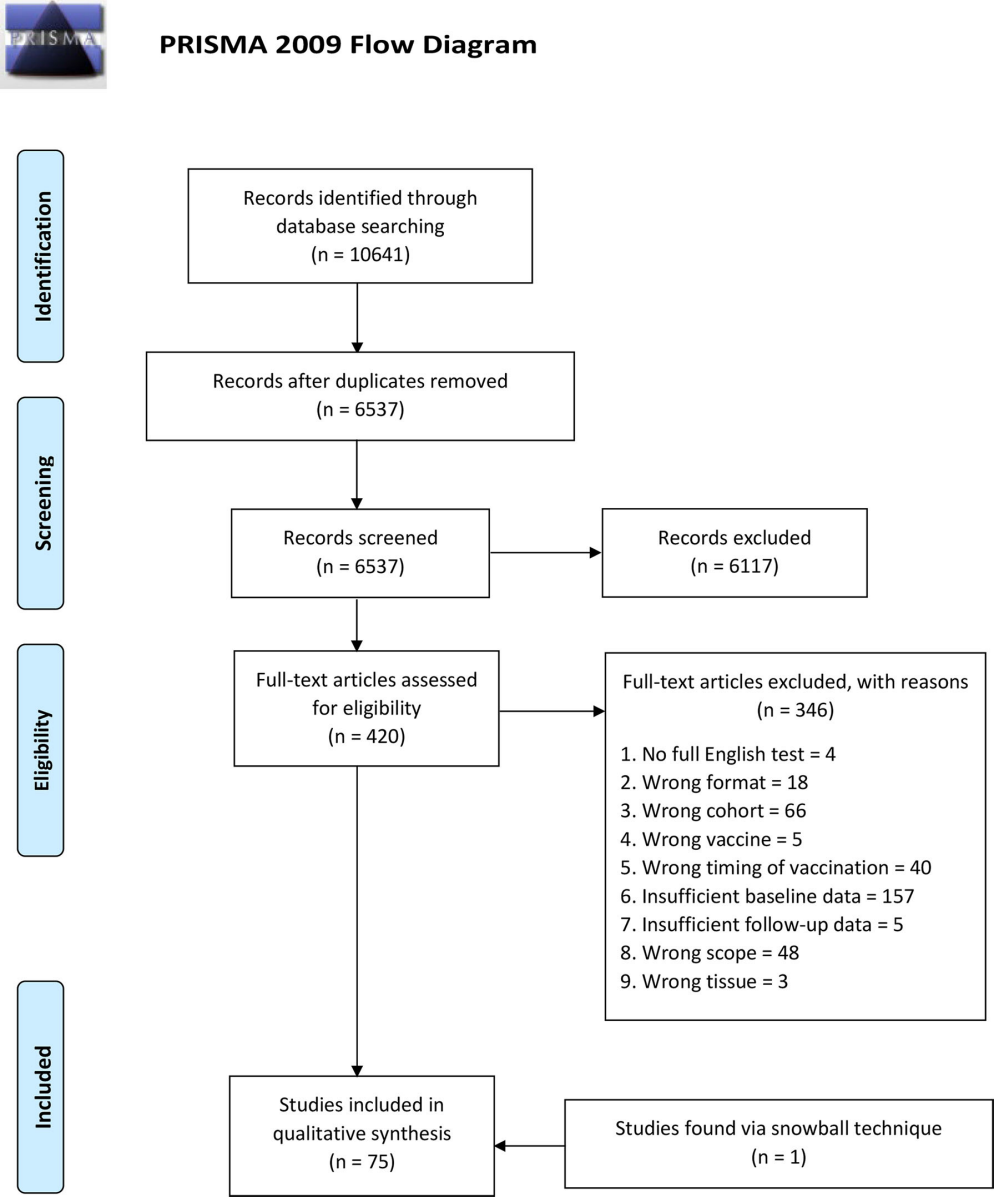
Prisma 2009 Flow Diagram. Overview of the screening process and article selection.

The literature search was performed on 16 December 2020 and yielded 10,641 results, of which 6,537 results remained after exclusion of duplicates. 6,117 articles were excluded based on title and abstract. The full text was retrieved for the 420 remaining articles (101 for extremities of life, 178 for immunodeficiencies, 142 for immunosuppression). After evaluation of the full text, 346 additional articles were excluded. One additional article was included using ‘snowballing’ technique. Thus, 75 articles (13 for extremities of life, 33 for immunodeficiencies, 29 for immunosuppression) were selected for final inclusion in this review (**Figure 2**). For three topics, (neonates/infant vaccination studies, vaccination studies in pregnancy and evaluation of the immune system using a neoantigen), a single or no article met the inclusion criteria. Therefore, these topics were not reviewed in this manuscript. To facilitate the reading, we summarized vaccines utilized in reviewed studies in **Supplemental Table 2**.

### Elderly

Multiple factors influence overall vaccine responsiveness in the elderly, such as decreased cell numbers, reduced B- and T-cell repertoire diversity, increased frequency of Ag-experienced cells, low-grade systemic inflammation and presence of exhausted immune cells (17, 21–23). In contrast to infants, in the elderly the immune system mostly relies on memory responses. For the elderly, pneumococcal and annual influenza vaccinations are recommended and responses to these vaccines are broadly discussed in literature. We included 13 publications evaluating vaccine responses to pneumococcal (polysaccharide) and influenza (protein) vaccines (**Supplemental Table 3**). In contrast to protein vaccines, polysaccharide vaccines trigger T-cell independent responses (15). Therefore, we have split the result sections of this review into polysaccharide, protein studies, and, when appropriate, polysaccharide-conjugate studies.

All studies in the elderly used younger adults as control group. However, between studies, the age difference between the elderly and controls differed from as little as 1 year (i.e. <65y/o and > 65y/o), up to 50 years. The age difference between groups is indicated in each study description (**Supplemental Table 2**). To exclude the impact of factors other than aging, we selected studies performed in individuals ≥64 years old in whom no or very limited comorbidities were reported. Studies focused primarily on pre- and post-vaccination differences between elderly and younger adults, and the elderly were in general presumed to be lower responders. Although information about the impact of baseline status on immune response could be retrieved, overall correlations with outcome variables were limited, especially in polysaccharide vaccine studies.

#### Responses to Polysaccharide Vaccines in Elderly

Six papers describing five different pneumococcal polysaccharide vaccine (PPV) studies in the elderly were included. In 2/5 studies, no significant differences were reported between the younger and older cohort regarding outcome variables (24–26). However, once only the oldest individuals were compared to the controls, significant differences were found in one of these studies (24, 25). Strictness of inclusion criteria varied between studies. Notably, the study reporting no differences between the elderly and controls, by Carson et al., had most stringent inclusion criteria. The overall risk of bias of the studies evaluating the response to PPV in elderly was low/moderate.

Baseline vaccine-specific Ig serum levels were heterogenous, with lower (27), comparable (26), or higher (25, 28) serum Ig levels in the elderly compared to controls. All studies reported increased vaccine-specific serum Ig levels post-vaccination in the elderly. For specific IgG serum levels, no differences between the elderly and controls were found. However, vaccine-specific IgM and/or IgA production was lower in the elderly post-vaccination (27, 28). Baseline avidity in the elderly seemed slightly higher, but was only commented on in one study (significantly higher), and post-vaccination avidity was comparable to (26) or stronger than (25) in controls. Lastly, opsonization capacity increased post-vaccination, but was lower compared to controls (25).

The (total) B-cell repertoire pre- and post-vaccination was evaluated by Kolibab et al. and Ademokun et al. When measured by spectratyping, clear changes were observed in total B-cell repertoire at d7 post-vaccination (after PPV and influenza vaccination) in both the elderly and controls (28). Nevertheless, at baseline and d28 the repertoire showed a reduced diversity in the elderly. Likewise, sequencing data indicated a lack of diversity even in absence of challenge (considerable clonality of the switched sequences). This was confirmed by Kolibab et al., who reported lower frequencies of somatic hypermutations and decreased oligoclonality in response to pneumococcal polysaccharides (PPS)4 and 14 (two PPV serotypes) in elderly (24). In the latter publication, the authors suggested for certain loci a possible association between gene utilization and antibody avidity.

Although Ig quantity, quality and diversity were well-covered, cellular aspects were evaluated to a lesser extent. In the elderly, total B-cell counts were lower (29) or comparable (25, 26) to controls, with one study showing a trend towards lower B-cell counts in the elderly. One study investigated B-cell subsets instead of total B-cell counts (27). Populations comprising primarily class-switched (CD20^+^CD27^+^IgD^-^) and non class-switched (CD20^+^CD27^+^IgM^+^IgD^+^) MBCs decreased with age (percentage and absolute cell counts). In contrast, naive B-cell percentages (CD20^+^CD27^-^IgD^+^) increased with age, although absolute cell numbers were reduced. *In vitro* stimulation of B cells resulted in lower Ig synthesis or plasma cell production in the elderly. Aside from this *in vitro* assay, only one study reported kinetics of (Ag-specific) B-cell subsets at d7 post-vaccination. Here, the authors found that Ag-specific B cells in controls were mostly CD27^+^IgM^+^ and in the elderly mostly CD27^+^IgM^-^ (29).

Overall, the PPV studies limitedly assessed the impact of B-cell status on vaccine responsiveness and focused primarily on differences between age groups. The elderly appeared to have lower Ig production, lower B-cell counts and reduced B-cell repertoire diversity, which may result in an overall lower vaccine responsiveness. Although no direct predictive factors were deduced from these PPV studies, it is noteworthy that the only study finding no differences between the elderly and controls had the most stringent criteria, possibly decreasing the biological age of the elderly cohort.

#### Responses to Protein Vaccines in Elderly

Eight studies evaluated responses to protein vaccines in the elderly; one to diphtheria/tetanus (DT) vaccine, and all others to influenza vaccine. All studies reported differences between the elderly and controls in at least one pre- or post-vaccination outcome variable. Although the overall risk of bias of the studies was low/moderate, the study by Nipper et al. had a serious risk of bias, mainly because of the data of this publication being comprised out of several separate study cohorts (30). Importantly, reporting methods differed between included publications, with some reporting fold changes and others reporting absolute values.

For influenza vaccination, multiple studies used neutralization or inhibition assays complementary to or instead of quantitative Ig detection (**Supplemental Table 2**). Only 3/8 studies reported a quantitative Ig readout. For baseline Ag-specific Ig serum levels, IgG was found to be lower compared to controls (26), and IgA higher (31). One study reported responses to influenza vaccination in three consecutive years and found in two years a higher and in one year a lower baseline Ig level in the elderly (32). All studies reported increased Ig levels post-vaccination, but serum IgG was consistently lower compared to controls (26, 31, 33). For IgM and IgA, serum levels were significantly (or tended to be) lower compared to controls too (31, 33). However, when comparing fold changes instead of absolute increases, no such differences were found for IgG (26).

Differences in neutralization or inhibition capacity (NIC) of antibodies pre- and post-vaccination were assessed in 6/8 studies. Three studies measured baseline antibody NIC in the elderly, which was lowered (34, 35) or comparable (33) to controls. Post-vaccination antibody NIC in the elderly was assessed more often, and was lower [fold change (33), titer (35)] or comparable [fold change (23), titer (31, 32, 34)] to controls. Interestingly, Abreu et al. found comparable inhibition titers in the elderly and controls, but reported a clear bias towards the H1HA vaccine component (present in vaccine for multiple years) over the H3HA component (updated annually) in the elderly, but not controls (32). Additionally, Kurupati et al. measured comparable neutralizing antibody levels, but reported a delayed increase and more rapid decay of these neutralizing antibody levels in the elderly (31).

In contrast to PPV studies, most protein vaccination studies evaluated cellular kinetics post-vaccination. Three studies reported total B-cell counts in the elderly, which were lower (30, 33) or comparable (26) to controls. In 6/8 studies baseline and/or post-vaccination kinetics of B-cell subsets were investigated. In general, MBC numbers (percentages or cells/10^6^ PMBCs) in the elderly were lower (23, 33, 35) or comparable (30) to controls. Again, naive B-cell percentages were higher (23, 33), but the naive B-cell count (per 10^6^ PBMCs) was lower in the elderly (35). In one study, phenotypic distinction between naive B cells and MBCs was doubtful; these counts were not included in this review (31). In the elderly, baseline plasmablasts percentages were lower (34) or comparable (30) to controls. Likewise, plasmablast expansion at d7 was lower (31) or comparable (33–35) to controls. Interestingly, Kurupati et al. found a lower plasmablast expansion in the elderly when using flow cytometry, but not when using ELISpot (31).

In addition, five studies explored specific age-associated B-cell phenotypes or differences in marker expression between the elderly and controls. Here, several unique phenotypes were evaluated, and in four studies correlations with vaccine responsiveness were reported.

Kurupati et al. reported reduced CD38 expression and loss of CD27 expression on part of the IgG producing cells (CD20^-^IgD^-^CD27^-^CD38^int^) in the elderly, and suggested this may be part of the immunosenescence process (31). Although no correlation with vaccine responsiveness was reported, the authors reported a delayed and less prolonged vaccine-specific Ig serum production in the elderly.

Next, Nipper et al. investigated vaccine responsiveness with a special focus on so-called age-associated B cells (CD21^-^T-bet^+^CD11c^+^) (30). Increased age was associated with expansion of atypical MBCs (CD10^-^CD20^+^CD21^-^CD27^-^) and reduced expression of PAX5 – a key regulator of B-cell identity and differentiation – and inhibitory molecules CD72 and CD85j on B cells. Additionally, lower PAX5 expression was associated with poorer vaccine responses. Lastly, a trend towards higher counts of atypical age-associated B cells (CD21^-^T-bet^+^CD11c^+^CD27^-^) and significantly lower resting MBC numbers (CD10^-^CD20^+^CD21^+^CD27^+^) were found in donors who responded poorly to vaccination.

Frasca et al. studied *in vitro* and *in vivo* responses to the monovalent pandemic (p)H1N1 influenza vaccine in the elderly (33). The percentage of class-switched MBCs at baseline correlated with the hemagglutinin inhibition response. Additionally, the level of AID expression at baseline (upon CpG stimulation) significantly correlated with *in vivo* vaccine responses. Thus, baseline AID expression and the percentage of class-switched MBCs may have predictive value for the vaccine responsiveness.

In another study, Frasca et al. reported the response to trivalent influenza vaccination (23). Here, the authors focused on the Senescent-Associated-Secretory-Phenotype (SASP), which may contribute to inflammaging. Significantly higher percentages of late/exhausted MBCs (CD27^-^IgD^-^) were found in the elderly. Within total MBCs, the late/exhausted MBCs showed the highest levels for many evaluated SASP markers. Class-switched MBCs (CD27^+^IgD^-^) positively correlated with *in vivo* responses, whereas late/exhausted MBCs showed a negative correlation. Thus, the percentage of late/exhausted MBCs may have predictive value for vaccine responsiveness.

Lastly, Kannan et al. studied responses to trivalent influenza vaccination, with focus on the anti-H1N1 response, and reported a trend towards lower numbers of transitional (CD20^+^IgD^+^CD27^+/-^CD38^+/-^), class-switched memory (CD20^+^IgD^-^CD27^+^CD38^-^) and double-negative B cells (CD20^+^IgD^-^CD27^-^CD38^-^) in the elderly (35). Moreover, the elderly had significantly lower baseline BTLA (B- and T-lymphocyte attenuator) expression on B cells, which inversely correlated with age. High BTLA expression on total mature B cells was linked to higher IgG and lower IgM vaccine-specific antibody responses irrespective of age. BTLA expression levels were linked to a better preservation of neutralizing antibody titers and improved recall responses. Lastly, the authors suggested that the decline in BTLA during immunosenescence may contribute to the lack of sustained antibody responses in the aged and their reduced ability to mount recall responses.

To summarize; multiple factors with potentially predictive value for the responsiveness to a protein vaccine (mainly influenza) were found in the elderly. For serum Igs, different patterns were observed for IgG, IgA, IgM or total serum Ig. Although different factors and B-cell subsets were evaluated, there was one clear predictor: the ‘age’ of the immune system. In 5/8 studies, a factor that was affected by age was evaluated, and donors with the least affected B cells showed better vaccine responsiveness. Although not surprising, these studies hint towards the assessment of the (baseline) immunological age as a predictor of vaccine responsiveness.

### Primary Immunodeficiency and Common Variable Immunodeficiency Disorders

Primary immunodeficiency disorder (PID) refers to a heterogeneous group of inborn disorders characterized by poor or absent function in one or more components of the immune system (36, 37). Individuals with a primary humoral immunodeficiency who meet specific criteria [e.g. ESID criteria (38)], among which markedly decreased IgG levels in combination with markedly decreased IgM or IgA levels, are classified as common variable immunodeficiency disorder (CVID) patients. Among 33 articles selected for immunodeficiencies, we included 9 studies performed in CVID patients (**Supplemental Table 4**). Studies in other PID patients were mostly case reports or did not meet other inclusion criteria, and therefore were excluded from this review.

Most (8/9) vaccination studies in CVID evaluated responses to polysaccharide vaccines. Goldacker et al. additionally evaluated five peptide vaccines and Gardulf et al. studied responses to Pandemrix (39, 40). Risk of bias was low in 8/9 studies and moderate in Gardulf et al. because of a substantial number of study drop-outs (9/57) (39). Most studies reported substitutive therapy, either with intravenous or subcutaneous Igs, while 3 manuscripts failed to provide such information (**Supplemental Table 4**). Only two studies indicated when samples were collected in relation to the treatment (39, 41).

Several classifications have been developed to describe the severity of phenotype in CVID patients (42). These classifications are either based on the ability to produce Igs upon stimulation *in vitro* (London), frequency of major B-cell subsets (Paris, Freiburg, EUROclass), or presence of defects in B-cell development (**Supplemental Table 5**) (43–46). Most of the here-described studies set out to determine to what extent classifications are predictive of vaccination responses.

#### Responses to Polysaccharide Vaccines in CVID

Yazdani et al. evaluated PPV responses in 25 patients and correlated vaccination outcome with four different CVID classifications. 22 (88%) patients were hyporesponsive (evaluated by the increase in vaccine-specific Igs), while 3 (12%) responded normally (42). Among responders two belonged to MB0 in Paris classification and group Ib in Freiburg classification, and one to Paris MB1 and Freiburg group II. In EUROclass classification, one responder was smB^-^21^low^ smB^-^Tr^norm^, one smB^-^21^norm^ smB^-^Tr^norm^ and one smB^+^ 21^norm^. Finally, two represented B-cell pattern 3 and one B-cell pattern 4 (**Supplemental Table 6**). Thus, classifications did not predict vaccination outcome. Similar conclusions were reached by Rezaei et al., who studied responses to meningococcal polysaccharide vaccine in 12 CVID patients (47). Out of seven responders (evaluated by serum bactericidal assay; SBA), two belonged to Freiburg group Ia, three to Freiburg group Ib, and two to Freiburg group II.

Goldacker et al. studied immune responses to PPV in 21 CVID patients (40). Here, both IgM and IgG responses to 10 PPSs were evaluated. Both IgM and IgG responses were identified in one patient from Freiburg group Ib (London A, Paris MB1) and one from Freiburg group II (London C, Paris MB1), only IgM responses were identified in one patient from Freiburg group Ib (London B, MB0 in Paris classification), and only IgG responses in one donor from Freiburg group II (London C, Paris MB2). In contrast to the above-mentioned studies, when the sum of all positive serotype-specific IgG anti-PPS reactions was correlated to the ‘Freiburg’ classification, a clear pattern emerged in favor of type II patients. This difference in conclusions seems to originate from a more detailed evaluation in the latter study. Moreover, only patients with normal IgM^+^ MBC percentages (>8%) produced anti-PPS antibodies. Thus, normal percentages of IgM^+^ MBCs appeared necessary, but not sufficient, for an efficient response.

Two bigger studies by Cavaliere et al. and Pulvirenti et al. also found an association between B cells and vaccination responses as evaluated by anti-PPS IgAs and/or IgMs (48, 49). In Cavaliere et al., 10/125 patients had detectable anti-PPS IgM and IgA, 25 only IgM and 2 only IgA. From all evaluated B-cell subsets, non-responders more often had reduced (<20 cells/ml) IgM^+^ MBCs (CD27^+^IgM^+^IgD^+^) and class-switched MBCs (<21 cells/mm^3^; CD27^+^IgM^-^IgD^-^). Since the relative distribution of B-cell subsets was not provided, direct translation to CVID classifications was impossible. A crucial role of class-switched MBCs in responses to PPV was further acknowledged by Ko et al. (50). Here, 53 patients were divided into Freiburg group I (33 patients) and II (20 patients). Group I mounted protective responses only against median 0.5/12 vaccine serotypes in contrast to 3/12 in group II.

In Pulvirenti et al., 14/74 patients were classified as IgA-responders (49). Non-responders had an increased frequency of naive B cells (CD27^-^CD21^+^CD38^+^) and a lower frequency of class-switched MBCs (CD27^+^CD21^+^IgM^–^) compared to responders, but no differences in other B-cell subsets. Interestingly, non-responders belonged to all three Freiburg classes (IA: 28%, IB: 56%, II: 16%), whereas the responders belonged to IB (27%) or II (73%) classes only. All responders belonged to the EUROclass smB^+^, while non-responders were B^-^ (9%), smB^-^ (36%), or smB^+^ (55%). Finally, long-lasting responders (seropositive after m36 ± 6) had a higher frequency of class-switched MBCs, in comparison to those, who lost their IgA response after the first assessment.

A few authors evaluated the impact of other markers on PPV responses in CVID. Yazdani et al., studied defects in signaling molecules, resulting in an aberrant B-cell composition, in 10 patients (41). Here, percentages of marginal zone-like (CD27^+^IgM^hi^IgD^+^) B cells, class-switched (CD27^+^IgM^−^IgD^−^) MBCs, non class-switched (CD27^+^IgM^hi^IgD^−^) MBCs, total MBCs and plasmablasts (CD19^low^CD21^int^CD38^hi^IgM^−(+)^) were significantly decreased in patients, which negatively correlated with the expression of apoptosis marker Annexin V. Furthermore, expression of phosphorylated (p) Akt was reduced in B cells of these patients and correlated with the level of PPV-specific antibodies. Sharifi et al. found that low numbers of end-stage MBCs and hyporesponsiveness to PPV in CVID patients were associated with higher expression of Toll-like Receptor (TLR) 2 on PBMCs (at baseline and upon stimulation) (51). This was further accompanied by lower mRNA expression for myeloid differentiation primary response 88 (MyD88) implying a defect downstream of TLRs.

#### Responses to Other Vaccines in CVID

Only two studies evaluated immune responses to other vaccine types in CVID. Gardulf et al. found that 8/48 CVID patients responded to monovalent influenza vaccine (39). At the time of CVID diagnosis, non-responders had significantly higher mean serum IgM levels and lower mean serum IgG1 levels than responders. No differences were found regarding absolute B-cell counts, but non-responders had less plasmablasts and more CD21^low^ B cells. Responders mostly belonged to EUROclass SmB^-^Tr^norm^21^low^ and B-cell pattern 5.

In addition to PPV, Goldacker et al. evaluated responses to five protein vaccines (40). Protective IgG responses against hepatitis A or B vaccines (HAV/HBV) were raised in 7 donors representing all groups in Freiburg (Ia:2, Ib:3, II:2), London (A:1, B:3, C:3) and Paris (MB0:3, MB1:2, MB2:3) classifications. IgG responses were rarely observed against recall DT vaccinations, and 6 patients responded to the Hemophilus polysaccharide–protein conjugated vaccine. When all positive IgG responses were considered, they were stronger in Freiburg group II. Again, most CVID patients with anti-protein vaccination responses had normal IgM^+^ MBC counts.

In conclusion, although CVID patients responding to vaccinations represented different groups in CVID classifications, seroconversion was most likely in patients with higher MBC percentages (>0.4%, consistent with Freiburg group II). This trend was clearer when vaccination responses were evaluated for different PPS serotypes and included analysis of IgMs and IgAs.

### Secondary Immunodeficiency - HIV

Depletion of CD4^+^ T cells in chronically HIV-positive individuals is accompanied by intrinsic B-cell defects e.g. accumulation of activated-mature B cells, exhausted ‘tissue-like’ B cells, and depletion of resting MBCs (52). Although nowadays these defects can be mostly reversed by early antiretroviral therapy (ART), this was not always the case in the past (53).

Here, we summarized 20 studies performed between 2000-2018 evaluating immune responses to PPV (n=6), influenza (n=9) and other protein vaccines (n=5). These studies were heterogenous regarding the design, size and age of the cohort, route of infection or duration of treatment (**Supplemental Table 4**). While in most studies all patients were on ART, bigger cohorts also included untreated individuals. All these factors need to be considered during results interpretation. Risk of bias was low in 13/20 studies and moderate in the remaining 7 (mostly due to confounding).

#### Responses to Polysaccharide Vaccines in HIV

First, only studies with polysaccharide vaccines will be discussed, and polysaccharide-conjugated vaccines will be discussed in the next sub-section. Results will be presented along four major topics: role of (I) classical and (II) atypical B-cell populations, (III) pre-existing B-cell memory, and (IV) B-cell repertoire.

There is no consensus regarding the role of baseline B-cell populations in PPV responses in HIV. From all classical B-cell subsets, IgM^+^ and class-switched MBCs are most frequently discussed. Tsachouridou et al. found that total and exhausted (CD19^+^CD21^low^CD27^-^) B-cell counts, but not MBC (CD19^+^CD27^+^) and IgM^+^ MBC (CD19^+^CD27^+^IgM^hi^) counts, at baseline correlated with vaccine-specific IgGs in patients four weeks post-immunization (54). In contrast, correlation between IgM^+^ (marginal zone) MBCs, and post-vaccination IgG serotype coverage and opsonophagocytic killing (OPK) activity was observed by Eisen et al. in vertically-infected HIV patients. Also Hart et al. found a positive correlation between baseline IgM^+^ MBC (CD19^+^CD27^+^IgM^hi^IgD^low^) numbers and post-vaccination anti-PPS IgMs (55, 56).

Several atypical B-cell subsets in HIV correlated with PPV responses. In ART-naive patients studied by Abudulai et al., the baseline proportions of CD21^low/-^ or BTLA^+^ B cells correlated negatively with IgG^+^ antibody secreting cells (ASCs) for three serotypes. However, in ART-treated patients, the proportions of CD21^low/-^ B cells correlated positively with the IgG^+^ ASCs to one serotype (52). A predictive role of CD21^low^ B cells was also shown by Eisen et al., who compared responses to PPV in vertically- and horizontally-infected HIV patients and controls (56). From the two patient cohorts, significant associations were found only in vertically-infected patients, who appeared more affected. CD21^low^ (CD38^low^CD21^low^) B cells correlated negatively with IgG serotype coverage and OPK activity, and anergic (CD27^-^CD21^low^) cell numbers correlated negatively with both IgM and IgG serotype coverage and OPK activity post-immunization. Of the dynamic markers, the strongest positive correlations were seen between CXCR5 expression (marker of cell trafficking) and IgM and IgG serotype coverage post-vaccination and serum OPK activity. Bcl2 expression (marker of apoptosis) correlated with post-vaccination IgG and IgM serotype coverage. No consistent relationship was seen between numbers of other B-cell phenotypes, CD95 and Ki-67 expression and vaccination outcome.

Two studies evaluated the impact of pre-existing immunological memory on immune responses to PPV. In Eisen et al., y1 IgG serotype coverage correlated with d0 IgM and IgG antibody responses, suggesting a predictive role of natural immunity (56). In contrast, Farmaki et al. showed no correlation between baseline anti-PPS IgM^+^ (CD19^+^CD10^−^CD21^hi^CD27^+^IgM^+^) and class-switched (CD19^+^CD10^−^CD21^hi^CD27^+^IgM^−^) MBC counts and anti-PPS IgG levels at m1 after PPV (57). Moreover, IgM^+^ MBCs were significantly reduced after vaccination.

Only one study, by Chang et al., set out to determine if differences in *IGHV* gene usage between HIV patients and controls can influence their responsiveness to PPV (58). While IgG^+^ B cells from HIV individuals frequently utilized *IGHV4* gene family, *IGHV3* and *IGHV5* were more abundant in controls. Upon vaccination, *IGHV3* was further expanded in controls, while patients showed more *IGHV5*.

Although not unanimous, studies in HIV point towards a positive correlation between IgM^+^ MBCs and PPV outcome. In contrast, presence of atypical B-cell subsets, especially CD21^low^, seems to be a negative predictor. The role of pre-existing immunological memory is ambivalent. While baseline antibody levels appear to be good predictors of long-term protection, this is not always the case for short-term cellular responses. T-independent antigens seem to drive pre-existing MBCs into terminal differentiation without replenishing the MBC pool. Further differences in *IGHV* repertoire between patients and controls, especially underrepresentation of *IGHV3* genes crucial for recognition of PPS, may further affect PPV responses in HIV.

#### Responses to Polysaccharide-Conjugated Vaccines in HIV

Three studies investigated correlations between B-cell parameters and vaccination outcome with conjugated polysaccharide vaccines. Johannesson et al. subdivided HIV patients into ART-responders, impaired ART-responders and ART-naive (59). ART-responders had more class-switched (CD27^+^CD38^-^IgD^-^IgM^-^) MBCs and more marginal zone-like (CD27^+^CD38^-^IgD^+^IgM^+^) B cells compared to impaired responders. Furthermore, ART-naive patients had more transitional (CD27^-^IgD^+^IgM^+/-^CD38^+^) B cells and plasmablasts (CD27^+^CD38^+^) than other groups. The concentration of marginal zone-like B cells, class-switched MBCs and plasmablasts correlated positively with post-PCV IgG concentrations, of which low concentration of class-switched MBCs was the strongest independent predictor of poor vaccine responsiveness.

Farmaki et al., who studied immune responses to PCV (and PPV 12 months later), quantified baseline levels of PPS-specific IgM^+^ (CD10^−^CD27^+^CD21^hi^IgM^+^) and class-switched (CD10^-^CD27^+^CD21^hi^IgM^-^) MBCs and correlated them with PPS-specific IgGs and class-switched MBCs after each vaccination (57). For PCV, such positive correlation was found between baseline PPS-specific IgM^+^ MBCs and both PPS-specific class-switched MBCs and anti-PPS IgGs.

Milagres et al. reported that both conventional and atypical B-cell subsets predicted vaccination responses to conjugated meningococcal vaccine (60). Exhausted B cells (CD27^−^IgD^−^CD21^−^CD38^+^) as well as short-lived plasmablasts (CD27^+^IgD^−^CD21^−^CD38^+^) were increased in ART-treated HIV patients and negatively associated with vaccine-induced SBA levels.

In conclusion, in contrast to PPV, PCV responses seem to mostly depend on baseline class-switched MBC levels, which is in line with the T-cell dependent character of B-cell responses elicited by PCV. Furthermore, upon T-cell dependent stimulation, PPS-specific MBCs seem to re-enter germinal centers and to differentiate into class-switched MBCs, which results in positive correlations between pre-existing immunological memory and vaccination responses. Finally, similar to PPV, abundance of atypical B-cell subsets correlates with poor vaccination responses.

#### Responses to Influenza Vaccines in HIV

Influenza viruses are associated with significant morbidity and mortality in HIV-infected children and adults (61, 62). Thus, several studies set out to determine the effect of abnormal B-cell maturation and activation in HIV on the magnitude, quality and memory of the immune response to various influenza vaccines.

Curtis et al. studied responses to monovalent influenza vaccination in HIV-infected children and adolescents (63). Among classical B-cell subsets, resting MBCs (CD21^+^CD27^+^) correlated positively and transitional B cells (CD21^-^CD27^-^CD20^-^) correlated negatively with B-cell memory (pH1N1 IgG ASCs) after the second vaccination dose. Additionally, transitional B cells correlated negatively with antibody avidity. Among atypical subsets, activated (CD38^+^HLADR^+^) B cells correlated negatively and activated immature B cells correlated positively with B-cell memory after second dose, while tissue-like B cells (CD21^-^CD27^-^CD20^-^) correlated negatively with antibody avidity. More responders were found in the older adolescents than in children, which was associated with pre-existing memory due to previous influenza encounters.

Rinaldi et al. investigated the impact of HIV and age on immune responses to (trivalent) influenza vaccine in adults (64). Surprisingly, young HIV patients showed severe signs of immunosenescence, i.e. increased frequencies of double-negative B cells (IgD^-^CD27^-^) and high expression of activation markers CD80 and PDL1 on B cells. Frequencies of CD80^+^ naive B cells correlated inversely with the H1N1 titer fold change, suggesting a negative impact of immune activation on vaccine responses in this group. Finally, baseline plasmablast frequencies positively correlated with H1N1-specific spontaneous ASCs at d7. Time under ART showed negative correlation with immunological abnormalities.

Other B-cell aberrancies observed in HIV such as increased expression of inhibitory receptor FcRL4 (65) or elevated frequencies of cycling (Ki-67^+^) and apoptotic (Annexin V^+^) B cells (66) did not correlate with influenza vaccination outcome.

Several studies showed that also early B-cell responses may discriminate between vaccination responders and non-responders. In two separate studies, Pallikkuth et al. evaluated immune responses to monovalent influenza vaccination in a small cohort of patients and controls (67, 68). Although the distribution of B-cell maturation stages at baseline differed between patients and controls, no differences were found between responders and non-responders among the patients. However, after vaccination only controls and responding patients showed an increased proliferation and expansion of MBCs and plasmablasts. Additionally, responders showed elevated levels of BAFF and APRIL (promotors of B-cell activation and Ig production), as well as IL-21 (secreted by CD4^+^ follicular helper T cells) upon vaccination. The latter was confirmed by Parmigiani et al. (65). When B-cell receptors for these soluble factors were evaluated, it turned out that at baseline non-responders had lower frequencies of BAFF-R and TACI-expressing MBCs than responders. After vaccination, BAFF-R^+^ B-cell frequencies decreased and TACI^+^ B-cell frequencies increased in controls and responding patients, but not in non-responding patients. H1N1 antibody titers correlated inversely with BAFF-R^+^ B cells and MBCs and positively with TACI^+^ B cells and MBCs at d28. IL-21R^+^ B cells were not significantly different between responders and non-responders at baseline, but increased at d28 in responders, which directly correlated with H1N1-specific antibodies. Moreover, cells from non-responding patients failed to respond to IL-21 in culture. Furthermore, Cagigi et al. found a reverse correlation between the baseline frequencies of IL-21R–expressing B cells, mature-activated (CD10^-^CD21^-^) and double-negative (CD27^-^IgD^-^) B cells, which were more frequent in non-responders (69).

Further studies by Cagigi et al. revealed hampered upregulation of AID in untreated HIV patients at m1 post-vaccination (70). Here, *in vitro* AID fold increase upon activation directly correlated to the *in vivo* anti-A(H1N1)pdm09 antibody increase at m1, and the maximum expression level of AID was significantly higher in individuals with protective antibody levels after six months. Thus, the ability of cells to upregulate AID can predict vaccination responsiveness.

Several studies investigated if immunological memory from previous antigen encounters can influence immune responses to influenza vaccine in HIV. Curtis et al. found that individuals who were seropositive at baseline (titer ≥1:40) had significantly higher IgG ASC levels upon monovalent influenza vaccination than seronegative participants (titer <1:40) (63). However, this was in contrast with Luo et al., who showed a reverse trend. Noteworthy, in the second study all participants had protective baseline Ab levels (titer >1:40), and responses seemed to reflect a plateau effect of vaccine-induced fold changes of antibody responses (66).

Wheatley et al. extended the evaluation of pre-existing memory by cellular analysis (71). Although patients and controls raised similar Ig levels upon vaccination, post-vaccination frequencies of MBCs against vaccine strains were significantly higher in controls. The magnitude of MBCs induced post-vaccination was proportional to their initial frequencies at baseline, while the impact of pre-existing serological responses varied for MBCs against different vaccine components.

In summary, extensive studies on influenza vaccine responses in HIV confirmed observations from previous vaccination models (positive role of MBC levels, negative role of transitional and exhausted/tissue-like B-cell levels) and extended them by demonstrating a (mostly) negative impact of activated B-cell subsets. Furthermore, here-reviewed manuscripts pointed at the potential role of *in vitro* studies (responses to IL-21, upregulation of AID upon stimulation) in predicting vaccination outcome. Regarding the role of pre-existing immunological memory, positive correlation between pre- and post-vaccination Ag-specific Ig levels seemed to exist only within a certain titer range and reaching protective Ig levels was not always accompanied by effective MBC responses. Finally, age, treatment and disease duration seemed to have an important modulatory role on top of HIV diagnosis.

#### Responses to Other Protein Vaccines in HIV

A few groups investigated immune responses to tetanus and hepatitis A or B vaccines in HIV. Only a limited number of correlations between baseline B-cell composition and vaccination responses was found. Weinberg et al. investigated primary immune responses to HAV in a large cohort of HIV-infected children on ART (72). From all investigated B-cell subsets, total B-cell percentage at baseline was the strongest predictor of vaccination responses (HAV antibody titer after second vaccination dose). Additionally, children who mounted HAV cell-mediated immunity (proliferative responses upon stimulation), had higher HAV antibody levels and MBCs percentage upon vaccination. In Van Epps et al., resting MBCs at baseline correlated with anti-tetanus Ig levels at w12 post-vaccination, while only a trend was found for HAV (53). Finally, Paris et al. showed no role of classical B-cell subsets, or CCR7^hi^CXCR5^hi,^or CCR7^hi^CXCR5^low^ B-cell subsets at baseline in predicting responses to HBV (increase in vaccine-specific IgGs) (73). Noteworthy, in these studies predictive role of MBCs was mostly found for recall, but not for primary responses.

### Responses to Vaccination in Asplenia

Asplenia can be an inborn condition or a result of surgical procedures e.g. due to an accident or underlying disease. In health, the spleen maintains a pool of MBCs and is a major source of IgM^+^ marginal zone-like B cells protecting against encapsulated bacteria (74). Therefore, most vaccination studies in asplenia focused on responses to bacterial polysaccharides. Among here-discussed studies, three had low, and one had moderate risk of bias.

Wasserstrom et al. studied patients splenectomized due to autoimmune conditions (n=19) and hereditary spherocytosis (n=6) vaccinated with PPV (75). In comparison to controls, both splenectomized cohorts had fewer MBCs (total, CD27^+^IgM^-^IgD^-^ and IgM^+^), which was statistically significant for the autoimmune group. Although splenectomized patients tended to have higher concentrations of anti-PPS IgGs at baseline (due to previous vaccinations), overall IgG responses upon immunization were comparable in patients and controls. In contrast, anti-pneumococcal IgMs were comparable at baseline, but less expanded in autoimmune patients upon vaccination. Despite an obvious defect in MBCs and IgM antibody responses in autoimmune patients, there was no direct correlation between anti-PPS IgG or IgM responses and CD27^+^IgM^+^ or class-switched MBC numbers.

Rosado et al. revealed differences in responses to PPV and PCV administered before and/or after splenectomy (76). 57 splenectomized adults and 11 children received PPV, and 10 children received PCV. Although splenectomy did not alter serum anti-PPS IgG concentration, the number of PPS-specific IgM^+^ and IgG^+^ MBCs was reduced in PPV-vaccinated individuals. Only in children, who received PCV after splenectomy, the number of PPS-specific IgG^+^ MBCs was similar to that of pediatric controls. Thus, PCV, but not PPV, administered after splenectomy could restore IgG^+^ PPS-specific MBCs.

Papadatou et al. investigated adults with β-thalassemia and asplenia vaccinated with PCV and in the past with PPV (77). PPS-specific IgGs were detected in all patients before and significantly increased upon vaccination. At baseline, all study participants had detectable IgM^+^ and IgG^+^ MBCs against at least one serotype. PPS-specific IgG^+^ MBCs, but not PPS-specific IgM^+^ MBC numbers, increased significantly upon vaccination. IgG^+^ and IgM^+^ MBC numbers, as well as IgG levels on d28, negatively correlated with number of previous PPV doses and positively with time since last PPV dose. This finding that multiple PPVs result in lower numbers of PPS-specific MBCs is in accordance with the hypothesis that the PPS antigens drive pre-existing PPS-specific class-switched MBCs into terminal differentiation without replenishing the MBC pool.

Finally, Giesecke et al. investigated tissue distribution of human Ag-specific MBCs, using responses to DT vaccine (78). In steady state, the spleen was the largest reservoir of tetanus toxoid (TT)–specific MBCs. After revaccination, controls, splenectomized and tonsillectomized individuals exhibited comparable emergence of anti-TT IgGs, TT-specific plasma cells, and TT-specific MBCs in blood. Moreover, molecular characteristics of TT-specific plasma cells were unaffected, despite reduced frequency of, mostly IgD^+^, peripheral blood MBCs in long-term splenectomized patients.

Overall, splenectomized patients seemed to have normal serological IgG responses to vaccination, but hampered IgM and MBC responses. IgG^+^ MBCs could be restored by protein, but not polysaccharide, antigens. Thus, proper evaluation of vaccine efficacy in splenectomized patients may require both serological and cellular analysis. Although no correlations were found between baseline B-cell subsets and vaccination outcome, it is important to realize that none of these studies was performed in patients with inborn asplenia, who present with a more severe phenotype.

### Immunosuppressive Treatment

Immunosuppressive treatment is prescribed for a variety of indications, e.g. autoimmune or malignant diseases, which by themselves already affect the immune system and vaccination responses. Therefore, it may be challenging to distinguish treatment effect from disease effect. However, certain study designs allow for this distinction, such as the inclusion of untreated or placebo-treated patients as controls. In such designs, any difference in vaccination response could be attributed to the only different condition: the administered treatment. Additionally, studies identifying immune patterns for vaccine (non-) responders are suitable for analysis in this review. However, when a study compares treated patients with healthy controls, it is difficult to distinguish treatment effect from disease effect. Therefore, results from these studies should be interpreted with caution. Nevertheless, they still provide valuable information on possibly predictive immune biomarkers for vaccination responses, and provide insight in the extent to which immunosuppressed individuals are able to mount normal immune responses. In addition to these challenges concerning study design, risk of selection bias is a very prevalent problem, as the start of treatment rarely coincides with the inclusion in the study.

In this section, we included all immunosuppressive agents or treatments directly or indirectly affecting (a part of) the B-cell compartment. We aimed to describe alterations in the B-cell compartment after immunosuppressive treatment, and how they correlate with attenuated vaccination responses. The 29 articles included for this topic have been subdivided according to type of treatment or indication: I) (allogeneic) hematopoietic stem cell transplantation (HCT) (n=6), II) rituximab (n=7), III) chemotherapy (n=7), IV) organ transplantation (n=5), and V) autoimmune diseases (n=6). In 7/29 studies, T-cell independent PPV responses were studied and in 27/29 studies T-cell dependent vaccination responses were studied. Due to limited inclusion of publications reviewing polysaccharide responses, they were summarized in one final paragraph. An overview of all included articles can be found in **Supplemental Table 7**.

#### Response to Protein and Protein-Conjugated Vaccines in Patients After Allogeneic Hematopoietic Stem Cell Transplantation (HCT)

Common indications for allogeneic HCT include malignant and non-malignant hematologic diseases. Prior to HCT, patients undergo a conditioning regimen (either myeloablative or non-myeloablative) that ablates the bone marrow, resulting in an immunocompromised state (79). Afterwards, immunosuppressive agents are often prescribed to prevent graft versus host disease (80). This results in a compromised immune system, that takes time to reconstitute (81). Six articles on the immune response in patients after HCT were included. These publications generally report heterogeneous study cohorts with both malignant and non-malignant indications, and a wide variety of immunosuppressive drug combinations, varying in mechanism of action and intensity.

Five articles that compared vaccination responses between patients and healthy controls frequently found a significantly poorer vaccination response in patients (82–86). Roll et al. distinguished responders and non-responders and found that non-responders were generally on a heavier immunosuppression regimen (87). In one study, patients with SCID (severe combined immunodeficiency) responded either poorer or similar to healthy controls, depending on the type of SCID (82). Here, vaccination outcome appeared primarily attributable to the type of underlying molecular defect, and not to any immune parameters.

However, in all other studies baseline immune parameters seemed predictive of vaccination responses in patients. Higher MBC numbers distinguished responders from non-responders in two studies (83, 87). Likewise, MBC (both class-switched and non class-switched) counts or percentages were lower in patients in two other studies (84, 85). Additionally, Avetisyan et al. found that patients had fewer influenza-specific B cells as measured by ELISpot at baseline (86).

Naive B cells discriminated responders from non-responders in one study (87). Another study found higher baseline plasmablast numbers in patients, whose response was poorer than healthy controls (84). However, in the latter study, the patient cohort was rather heterogeneous regarding indication for HCT, graft versus host disease occurrence, and immunosuppressive treatment.

In summary, higher levels of MBCs (class-switched and non class-switched) in this cohort are likely to correlate with a better vaccination response. The same might hold for a higher frequency of naive B cells, influenza-specific B cells, and a lower frequency of plasmablasts.

#### Response to Protein and Protein-Conjugated Vaccines in Rituximab-Treated Patients

Rituximab specifically depletes CD20^+^ B cells, which means that any vaccination studies regarding B cells can be performed only after at least partial B-cell recovery (88). Seven studies reporting the effect of rituximab on the immune response were included.

Since rituximab is often administered in addition to other immunosuppressive agents, in a variety of conditions, it is difficult to isolate rituximab treatment effects. One placebo-controlled study in rituximab-treated patients reported attenuated responses to a neoantigen, but not to recall antigens (89). However, another placebo-controlled study did find attenuated responses to recall antigens in rituximab patients, as did four other studies with a slightly less suitable design (90–94). Only one study did not find differences in vaccination responses between patients and healthy controls, although baseline B-cell profiles were very similar in this study (95).

In this group, a critical role for MBCs was established as well. Remarkably, all studies found that MBC counts or percentages were reduced in rituximab-treated patients compared to controls (89–95). Three of them were however not able to correlate this lack of MBCs to a poorer antibody response: Puissant-Lubrano et al. and Pescovitz et al. found that MBC counts did not distinguish responders from non-responders, while Cho et al. found normal antibody responses despite a lack of MBCs in patients (89, 92, 95). As patient antibody responses were relatively normal in these studies, they might have been insufficiently powered to detect these correlations.

Although MBCs did not reconstitute in any study, some studies reported naive B-cell reconstitution. Two studies reported low naive B-cell counts in combination with poorer antibody responses. One of them found that higher naive B-cell counts distinguished responders from non-responders (91). Cho et al. reported higher transitional B-cell percentages in patients, although antibody responses were not different from healthy controls (95).

Additionally, some singular findings were reported. Pescovitz et al. reported that in a neoantigen vaccination setting, rituximab impairs class-switching up to the first booster vaccination (89). Nazi et al. found that rituximab patients had more plasmablasts than placebo-controls, while they responded poorer to vaccination (90). Higher total B-cell numbers were found by Eisenberg et al. to distinguish responders from non-responders (91).

In summary, limited correlations between baseline B-cell parameters and vaccine responses were found in this cohort, but that might be due to heterogeneity of additional immunosuppressive treatment within study cohorts. In general, it can be concluded that rituximab hampers antibody responses to neoantigens and recall antigens, and that higher MBC numbers are likely correlated with a better antibody response. Additionally, there might be a predictive function for higher total or naive B-cell counts, and lower plasmablast counts.

#### Response to Protein and Protein-Conjugated Vaccines in Patients Treated With Chemotherapy

Although chemotherapy can consist of different agents, intensities, and durations, a common side effect is immunosuppression (96). Seven studies in patients using chemotherapy were included. In these studies inclusion of a placebo treatment would be unethical. Regardless, differences within patient groups or between responders and non-responders might still be of value in our analysis. One study did use a T-cell dependent influenza vaccine, but only assessed T-cell responses to this antigen and is therefore not included in this section (97). It is however included in the polysaccharide section, as it did evaluate B-cell responses for PPV.

Two studies reported adequate vaccine responses in immunosuppressed acute lymphoblastic leukemia (ALL) patients compared to controls (98–100). However, Ek et al. reported that high-risk patients, who received the heaviest immunosuppression, were less successful in mounting a memory response (99). Two other studies, in either AML patients or ovarian cancer patients, reported lower geometric mean titers or seroconversion rates than in healthy controls, although their patient cohort was rather heterogeneous regarding immunosuppression intensity (101, 102).

Kersun et al. also performed a study in ALL patients, and found that patients vaccinated during induction therapy instead of later phases of chemotherapy had higher MBC counts, likely correlating with an increase in influenza-specific antibody titers (r=0.19) (103). Although Koskenvuo et al. described a similar lack of MBCs in ALL patients, they did respond normally to PCV (98). Another study, in AML patients, found that MBC counts were significantly higher in responders (104). This was corroborated by Reilly et al. who reported lower MBC counts in AML patients compared to controls, in combination with a poorer antibody response (102). Chu et al. reported lower MBC counts in ovarian cancer patients as well, in combination with lower seroconversion rates than in healthy controls (101).

Subnormal levels of naive B cells in patients were reported only by Koskenvuo et al. and Reilly et al., with the former reporting normal antibody responses and the latter poorer antibody responses (98, 102). On the other hand, total B-cell counts might be predictive of influenza-specific responses in patients using chemotherapy. Kersun et al. reported a positive correlation between total B-cell counts and antibody titers (r=0.23) (103). Two other studies described a lack of total B cells in combination with a lower antibody response (101, 102). However, in a PCV setting, Koskenvuo et al. reported reduced total B-cell numbers in patients, but normal antibody responses (98).

Singular findings were reported in this group as well. Ek et al. reported normal vaccine responses despite low CD5^-^ B-cell counts in ALL patients (99, 100). Goswami et al. ran an extensive flow cytometry panel and reported decreased CD86^+^ B-cell populations, but increased transitional B cells, in non-responding AML patients compared to healthy controls (104).

In conclusion, it is difficult to separate disease and treatment effects in patients on chemotherapy. ALL patients appear to respond rather well to vaccination. However, in an influenza vaccination setting higher total B-cell and MBC counts distinguished responders from non-responders.

#### Response to Protein and Protein-Conjugated Vaccines in Patients With Post-Transplantation Immunosuppression

After solid organ transplantation, immunosuppressive therapy is often administered to avoid graft rejection. In this case, ‘disease effect’ is negligible, as the act of transplantation itself is unlikely to be the cause of altered vaccination responses, although an immune reaction against the graft might occur. Therefore, valid inferences can still be made in less strict designs, such as comparing patients to healthy controls. However, immunosuppressive treatment might still be very heterogeneous among patient cohorts. Five post-transplantation studies were included.

Struijk et al. found that kidney transplant patients were unable to mount a response against neoantigen immunocyanin, whereas healthy controls could (105). The mycophenolate sodium (MPA)-treated group was unable to mount a recall response to TT, but other patient groups (treated with cyclosporine or everolimus) were, despite varying levels of pre-existing Ag-specific IgG levels. Cowan et al. and Egli et al. also described a poorer response in patients than in healthy controls (106, 107). The other two studies, although heterogeneous regarding cohort constitution (underlying condition and treatment), found contradictory results as their patient cohorts responded comparably to healthy controls (85, 92).

MBCs were reduced in patients in two studies, although this coincided with a poorer antibody response in only one study (92, 105). However, the other study used a more heterogeneous cohort (92). Similarly, naive B cells were reduced in patients in two studies, of which one reported a poorer response (85, 105). Again, the study reporting normal responses had a heterogeneous patient cohort (85).

Total B-cell numbers, which were reduced in patients, correlated to the anti-TT response in Struijk et al. (r=0.38) (105). A reduced number of total B cells was also found in Puissant-Lubrano et al. (92). In other studies, a few singular findings were reported. Cowan et al. reported low plasmablast numbers in patients, combined with poor ELISpot and antibody responses (106). Egli et al. found that patient-responders had a significantly higher HLA-DR and CD86 expression at baseline across all B-cell subsets (107). Puissant-Lubrano et al. reported normal antibody responses in patients, despite lower CD5^+^ MBC numbers (92).

The heterogeneity of here-described studies hampered identification of strong vaccination outcome predictors. In a randomized trial, Struijk et al. concluded that patients on post-transplantation immunosuppression were unable to mount normal humoral vaccination responses to neoantigens. Higher total B-cell numbers and plasmablasts might contribute to an adequate recall response, as well as activated B-cell subsets.

#### Response to Protein and Protein-Conjugated Vaccines in Autoimmune Patients Using Immunosuppression

In contrast to transplantation settings, the disease impact on vaccination responses cannot be ignored in autoimmune diseases, in which the immune system is considered abnormal even without immunosuppressive treatment. Six studies described such cohorts, although one assessed only PPV responses and is therefore not included in this section (108).

Vaccination responses in patients were attenuated in two studies (109, 110), and normal in two others (111, 112). Noteworthy, the latter study cohorts were very heterogeneous regarding immunosuppressive treatment.

In general, baseline B-cell parameters were less predictive of vaccination responses in this group. Bingham et al. reported higher MBC percentages and better vaccination responses in BAFF-inhibited patients compared to methotrexate-only patients (113). However, Salinas et al. reported higher amounts of non class-switched MBCs in anti-TNF-treated patients, who responded worse than healthy controls (110). Although Kamphuis et al. found lower amounts of MBCs in patients than in healthy controls, vaccine responses were normal (112).

More naive and immature B cells were found in the poorer responding methotrexate-only group by Bingham et al. (113). On the other hand, Salinas et al. reported lower naive B-cell numbers in patients who responded poorer than healthy controls (110).

ELISpot data at baseline did not correlate to vaccination responses in patients with autoimmune diseases. Kobie et al. reported similar baseline numbers of influenza-specific cells between patients and controls, although vaccination responses (both humoral responses and plasmablast induction) were worse in patients (109). Neither did Heijstek et al. find any correlation between baseline ELISpot data and vaccination responses (111).

Salinas et al. furthermore reported an impaired degree of somatic hypermutation in B cells of anti-TNF-treated patients, who responded poorer to vaccination than healthy controls to neoantigen HBV (110). Kamphuis et al. performed extensive flow cytometry, and found that although MBCs and natural effector B cells were significantly reduced, nearly all patients mounted adequate antibody responses (112).

In summary, patients with autoimmune diseases who use immunosuppression tend to mount impaired responses to vaccination. Naive and immature B-cell counts or ELISpot data did not predict vaccination responses. Also, MBCs appeared less predictive in this patient group. Impairment in somatic hypermutation might attenuate neoantigen responses.

#### Responses to Polysaccharide Vaccines in Immunosuppressed Patients

Seven studies in immunosuppressed patients investigated PPV responses, of which one in chemotherapy, one in post-transplantation, and five in autoimmune patients.

In 5/7 studies, an attenuated humoral anti-PPV response in patients was reported, compared to either healthy controls or untreated patients (90, 97, 105, 108, 110). A normal response was found in rheumatoid arthritis patients by Bingham et al., who used a randomized placebo-controlled design (113). Kamphuis et al. also found normal vaccination responses in sarcoidosis patients compared to healthy controls, but the treatment variety in this cohort might have been too large to detect differences in vaccination responses that could be attributed to immunosuppressive treatment (112).

The effect of chemotherapy on PPV responses was evaluated by De Lavallade et al. in chronic myeloid leukemia (CML) patients on a tyrosine kinase inhibitor (97). Median pre-vaccination anti-pneumococcal IgG levels were higher in patients, whereas anti-pneumococcal IgM levels were lower both pre- and post-vaccination. Responding patients had significantly more non class-switched and class-switched MBCs than non-responders. Non class-switched MBCs positively correlated with anti-pneumococcal IgM titers post-vaccination.

Struijk et al. evaluated a cohort of immunosuppressed kidney transplant recipients (105). Total, naive and memory B cells were lower in patients than in healthy controls, and lowest in MPA-treated patients. Pre-vaccination PPV-specific IgG levels were lowest in MPA-treated and cyclosporine-treated patients, who both responded significantly worse to PPV than healthy controls, whereas the everolimus-treated group was able to mount a normal response.

In both studies, MBCs appeared predictive of a humoral anti-PPV response. High quality evidence for a predictive role of total and naive B-cell numbers was provided by Struijk et al., who conducted a randomized controlled trial.

Five studies in patients with autoimmune diseases were included, but the heterogeneity in immunosuppressive treatment is rather extensive. A major limitation of these study cohorts is that the disease effect on vaccination responses cannot be ignored, and only placebo-designs or within cohort comparisons might allow for valid inferences.

Pre-vaccination Ig levels were assessed by all studies, of which only two compared these levels to controls (110, 113). These two studies did not find any significant differences in pre-vaccination IgG titers between untreated/placebo-treated and treated patients.

Regarding cellular data, 4/5 studies reported absolute baseline B-cell counts, and 2/5 studies reported relative B-cell subset percentages. Higher baseline naive B-cell counts were associated with a higher post-vaccination titer in two studies (90, 110). One study, in which similar vaccination responses were mounted despite a difference in naive B-cell percentages, contradicted this result (113).

MBCs appeared less predictive of anti-PPV responses in immunosuppressed autoimmune patients. One study reported lower MBCs in combination with lower vaccination responses (90), whereas two reported a difference in MBCs but not in vaccination responses (112, 113), one reported higher non class-switched MBCs in combination with lower vaccination responses (110), and one reported higher class-switched MBCs in combination with lower vaccination responses (108).

One study reported that higher plasmablast amounts were associated with lower vaccination responses (90). Another reported impaired somatic hypermutations in anti-TNF-treated patients, in whom vaccination responses were also poorer (110).

Kamphuis et al. studied many B-cell populations by flow cytometry (112). However, the studied sarcoidosis patient group was highly heterogeneous regarding type and intensity of immunosuppressive treatment, making it difficult to draw clear conclusions regarding the predictive value of B-cell baseline data. They found significantly reduced numbers of IgM^+^, IgG^+^, and IgA^+^CD27^+^ MBC, CD21^low^CD38^low^ anergic B cells and natural effector B cells, whereas CD27^−^IgA^+^ B-cell numbers were significantly increased in patients. Despite these differences, nearly all patients managed to mount an adequate antibody response to all vaccines.

To summarize, this limited amount of evidence shows that it is likely that immune responses to PPV are mainly determined by naive B cells, and to lesser extent by MBCs. Pre-existing immunity appears to be of inferior importance in PPV responses.

## Discussion

Here we reviewed 75 manuscripts that evaluated the impact of baseline B-cell status on immune responses to vaccination with the ultimate goal to identify parameters predictive of vaccine efficacy. In our search, we covered a range of conditions within three major topics: extremities of life, immunodeficiency and immunosuppression.

Despite a comprehensive literature search, we were unable to compare studies on vaccination responses in pregnancy and infants as for both topics only one study fulfilled inclusion criteria (85, 114). Similarly, no studies in PIDs or using vaccination with neoantigens to evaluate the immune system fulfilled the inclusion criteria. Lack of baseline information, wrong study cohort and wrong study scope were the most frequent reasons for exclusion. Furthermore, several studies were excluded because they did not report results of a control group, therefore preventing objective assessment of B-cell aberrancies and their correlation with vaccination responses.

**Figure 3:**
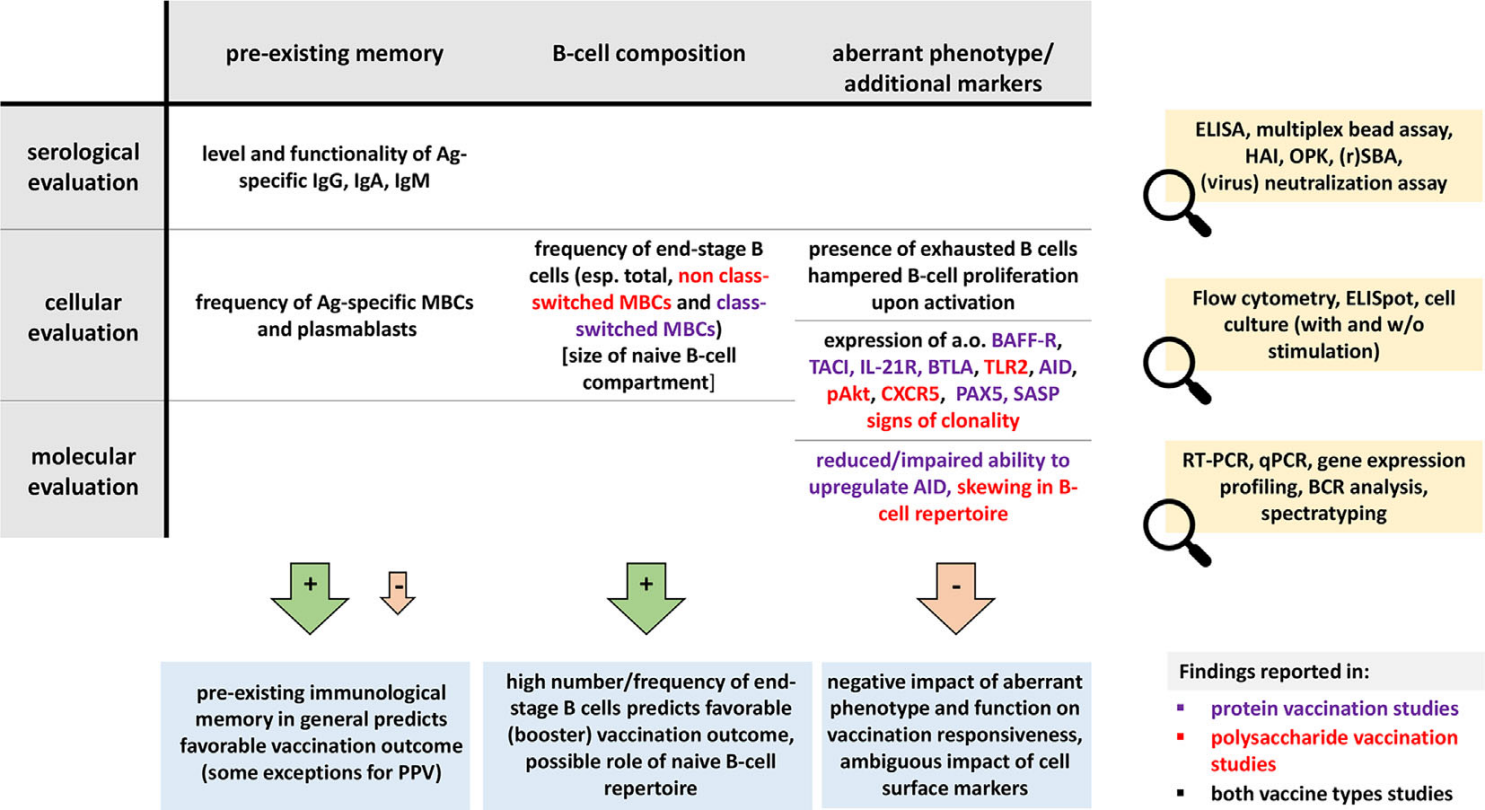
Overview of B-cell parameters predictive of vaccine efficacy. The different evaluation levels (serum-, cell- or molecular-based) are indicated in the rows, whereas the different B-cell parameters are indicated in the columns. Proposed detection techniques are shown in the yellow boxes. Blue boxes contain the general conclusions per parameter. Positive impact is indicated with green arrows (and a plus-symbol), while negative impact is indicated with red arrows (and a minus-symbol). In the ‘B-cell composition’ column, colors show general trends in polysaccharide and protein vaccines, whereas in the ‘aberrant phenotype/additional markers’ column, colors indicate in which vaccination settings markers were evaluated. PPV, pneumococcal polysaccharide vaccine; ELISA, Enzyme-Linked Immunosorbent Assay; HAI, hemagglutinin inhibition; OPK, opsonophagocytic killing; SBA, serum bactericidal assay; ELISpot, Enzyme-linked ImmunoSpot; w/o, without; esp., especially; RT-PCR, reverse-transcriptase polymerase chain reaction; RT qPCR, real-time quantitative polymerase chain reaction; BCR, B-cell receptor.

Extremities of life were represented by studies in the elderly, immunodeficiencies by CVID, HIV and asplenia, and the immunosuppressed group was relatively diverse. Noteworthy, studies in the elderly were excluded if any (major) comorbidities were mentioned. Although this approach was the most reliable to evaluate the impact of aging, data may not be fully representative of a general aging population in which co-existing diseases are frequent and influence the biological age. On the contrary, in other evaluated conditions, we included patients from different age groups. Since age, disease duration and type of medication may influence vaccination responses, we reported these parameters whenever they could influence data interpretation. Direct comparisons between publications were hampered by differences in population definitions, use of different vaccine efficacy readouts, but also ways of reporting the data (absolute numbers (cells or units/mL), absolute increases and fold changes). Although not the scope of this study, these differences would hinder meta-analysis. Overall, we found several B-cell factors influencing vaccination responsiveness with a potential for vaccine efficacy monitoring. Three topics spanned all sections: (I) lack of or reduction in end-stage B cells, (II) presence of phenotypically aberrant cells, and (III) impact of pre-existing immunological memory to a given antigen. In **Figure 3** we summarize parameters and assays which may have a predictive role in evaluating vaccination efficacy in immunocompromised individuals.

Overall, reduced MBC numbers prior to vaccination seem to be the most common predictor of poor vaccination outcomes (23, 27, 30, 40, 48–50, 55, 59, 83, 85, 91, 93, 94, 97, 102–104, 110). Depending on the situation, this role can be assigned to total, non class-switched MBCs or class-switched MBCs. While non class-switched (IgM^+^, marginal zone-like) MBCs have been shown to be involved in responses against polysaccharides, their ambivalent role in-between the studies may be attributed to diverse phenotypical definitions of this subset or heterogeneity of study cohorts (97, 115). Although MBC numbers are mostly used to classify CVID patients, low MBC numbers are also observed in chronically infected HIV patients and in patients using immunosuppression. In the latter case, MBC numbers often remained low, even if naive and total B-cells had reconstituted already, and patients struggled to mount adequate vaccination responses. Unfortunately, a clear cut-off value, above which vaccination responses would be successful, is difficult to define. The range of MBC numbers or percentages in vaccine responders and non-responders largely overlapped. Studies in CVID revealed that some patients can undergo seroconversion with <0.4% MBCs. These low numbers of MBCs are hardly ever observed in other conditions. Moreover, impaired somatic hypermutation processes or class-switching may hamper vaccine responses as well (24, 89, 110).

Although only a limited number of primary vaccination studies have been included in this review, primary vaccination responses rather depend on total or naive B-cell numbers as this is the pool from which Ag-specific cells are recruited (72, 105, 110).

Several atypical B-cell subsets have been described to correlate with poor vaccination responses. These were mostly classified either as exhausted or activated and were frequently described during aging, immunodeficiencies and autoimmunity (23, 30, 31, 52, 53, 56, 63, 64, 112, 116). Again, phenotypic description of exhausted/tissue-like MBCs differed in-between studies, but low expression of CD21 and/or simultaneous lack of CD27 and IgM/IgD were frequently described (**Supplemental Table 1**). A study in elderly reported that these cells had reduced proliferation and effector functions, were transcriptionally and metabolically active, and secreted pro-inflammatory cytokines (23).

Immunological memory exists in the form of Ag-specific MBCs, antibody-secreting plasma cells and their products, antibodies. In the majority of recall vaccination studies with protein antigens, levels of Ag-specific antibodies and MBCs at baseline seemed to positively correlate with vaccination outcome, at least within a certain titer range (55, 56, 58, 59, 66, 86). These pre-existing Ag-specific Igs and MBCs seemed to reflect the ability of the immune system to generate protective responses. Additionally, upon consecutive antigen encounters, Ag-specific MBCs from previous responses re-enter germinal centers to replenish the MBC pool. This situation is more complex for vaccination with polysaccharide antigens, when re-stimulated Ag-specific MBCs seem to undergo terminal differentiation without replenishing the MBC pool (57). Papadatou et al. even found a negative correlation between previous PPVs and immune responses to PCV (77). In line with this, Musher et al. reported that individuals who received PPV first and PCV later, responded worse than individuals who received PCV first and PPV later (117). Noteworthy, the ability to produce protective antibody titers is not always reflecting the ability to effectively generate MBCs, e.g. in splenectomized patients, MBC formation may be hampered despite unaffected antibody responses. Thus, both parameters should be measured to reliably assess vaccination outcome (76, 77). Conversely, whether the presence of MBCs in the absence of presumably protective antibody titers is sufficient for effective responses upon re-exposure is a viable question in the light of current SARS-CoV-19 pandemics (118).

In line with the presence of exhausted B cells, in multiple vaccination studies in elderly and HIV patients, individuals with more signs of immunological aging had lowered vaccine responses. Thus, baseline assessment of immunological age may predict vaccine responsiveness. Aside from the already discussed readouts, other methods may be used as alternative or complementary assays, such as the in-depth assessment of the B-cell repertoire or the kappa-recombination excision circles (KRECs) to determine the number of cell divisions that the naive B-cell population has undergone (13). Likewise, *in vitro* assays performed on fresh or frozen material, as performed in some of the reviewed publications, may give an impression of overall B-cell responsiveness (68, 70). Alternative assessments could include evaluation of B-cell responsiveness upon stimulation, e.g. by analysis of calcium-flux or phosphorylation of signaling molecules. However, these analyses require proper standardization and generation of reference data (12, 16, 119).

While certain parameters (frequency of MBCs, presence of exhausted B cells, pre-existing memory) were evaluated for several vaccine types, other characteristics were only studied in one specific model. Therefore, it is difficult to assess to which extent these characteristics are generally applicable for monitoring. Still, we identified an interesting set of markers, of which the utility for predicting vaccine responsiveness may be further investigated. Again, while certain correlations were shared by different patient groups, we also found conflicting findings. One example is the baseline frequency of activated B cells, which were positively correlating with vaccine responses in two studies involving immunosuppressed patients, but frequently negatively correlating with vaccine responses in HIV patients. Unification of markers used to define B-cell activation status might verify such contrasting findings.

Most vaccinations are administered early in childhood and boosted later in life. Since here-reviewed studies were mostly referring to booster responses, their heavy dependence on pre-existing MBCs is not surprising. As mentioned in several reviewed articles, these types of responses can be influenced by so called original antigenic sin, which implies that the development of immunity against pathogens/Ags is shaped by the first exposure to a related pathogen/Ag (120). We, and others, have recently observed that the type and magnitude of an immune response to a pertussis booster vaccine is heavily dependent on the type of priming and stronger in case of a whole cell vaccine as compared to an acellular vaccine (Diks et al., manuscript submitted) (121–123). Additionally, in case of e.g. respiratory pathogens, natural encounter results in IgA responses, which can be boosted upon vaccination (12, 124). In this case, evaluation of IgA responses may complement the detection of IgG responses as vaccine read-out (32, 48, 49, 124). This may be even more relevant for (alternative) vaccination routes, e.g. administration via mucosal surfaces.

In this review, we focused on the role of B-cell status in predicting vaccine efficacy. However important, prediction of vaccination safety may be even more crucial. Despite comprehensive literature search, we did not find any studies evaluating live-attenuated vaccines in immunocompromised individuals. This is understandable for ethical considerations. However, it is reasonable to assume that vaccination with live-attenuated vaccines will be safer in these patients who generate effective responses to other vaccine types. Although we intended to include a section on the use of neoantigen vaccination to define B-cell defects and thus predict vaccination responses, we were unable to include any publications on this subject. However, we believe that this is an important topic for future research, with clear clinical applications. Vaccination with a neoantigen can provide in-depth insight into both quantitative and qualitative B-cell defects. The main advantage of using a neoantigen over recall vaccine antigens is the ability to assess primary immune responses next to booster responses. The immune response to neoantigens would not rely on the remaining immunological memory from previous encounters, but fully depend on the current status of the immune system. In this way, a comprehensive overview of the functionality of the immune system can be obtained, treatment can be personalized, and vaccination responses can be predicted.

Despite covering a broad range of conditions, we are aware that the current review does not exhaust the topic. Multiple other factors can have an impact on B-cell composition and therefore vaccination responsiveness. These can be other autoimmune diseases, latent viral infections (e.g. EBV, CMV), alcoholism, but also less obvious factors such as gender, race, the season of the year, psychological stress or nutritional status (125–133). Neither covered by the scope of the review are all aspects of T-cell and innate cell immunity, which may severely influence vaccination responsiveness. Nevertheless, we believe that the selection made here shows the major trends and will inspire new studies on B-cell monitoring for better evaluation of vaccine efficacy.

## Data Availability Statement

The original contributions presented in the study are included in the article/**Supplementary Material**. Further inquiries can be directed to the corresponding author.

## Author Contributions

AD, LO, and MB performed the literature search, screening of the articles and wrote the manuscript. LL performed screening of the manuscripts. LS, HJ, LV, and JD provided feedback on the article. All authors contributed to the article and approved the submitted version.

## Funding

AD, MB, and JD are involved in the PERISCOPE project. The PERISCOPE project has received funding from the Innovative Medicines Initiative 2 Joint Undertaking under grant agreement No 115910. This Joint Undertaking receives support from the European Union’s Horizon 2020 research and innovation program and EFPIA and BMGF. LO received an MD/PhD scholarship from the LUMC (Leiden, Netherlands).

## Conflict of Interest

AD, JD, and MB report inventorship of the patent “Means and methods for multiparameter cytometry-based leukocyte subsetting” (NL2844751, filing date 5 November 2019), owned by the EuroFlow Consortium (134). In addition, JD reports to be chairman of the EuroFlow scientific foundation, which receives royalties from licensed patents, which are collectively owned by the participants of the EuroFlow Foundation. These royalties are exclusively used for continuation of the EuroFlow collaboration and sustainability of the EuroFlow consortium. Lastly, JD reports an Educational Services Agreement from BD Biosciences (San José, CA) and a Scientific Advisor Agreement with Cytognos, all related fees and honoraria go to LUMC.

The remaining authors declare that the research was conducted in the absence of any commercial or financial relationships that could be construed as a potential conflict of interest.

## Publisher’s Note

All claims expressed in this article are solely those of the authors and do not necessarily represent those of their affiliated organizations, or those of the publisher, the editors and the reviewers. Any product that may be evaluated in this article, or claim that may be made by its manufacturer, is not guaranteed or endorsed by the publisher.
