# Supplementary material for: B-Cell Immunophenotyping to Predict Vaccination Outcome in the Immunocompromised - A Systematic Review
Source: Front Immunol. 2021 Sep 7;12:690328. doi: 10.3389/fimmu.2021.690328 (PMC8452967; doi:10.3389/fimmu.2021.690328)
Supplement: Supplementary file 1 [file DataSheet_1.docx]

**Supplemental information- Supplemental Text 1**

**Search strategy used for PubMed database**

("B-Lymphocytes"[Mesh] OR "B-Lymphocyte*"[tw] OR "B cell*"[tw] OR "B-cell*"[tw] OR "B lymphocyte*"[tw] OR "B-lymphocyte*"[tw] OR "plasma cell*"[tw]) AND ("Vaccination"[Mesh] OR "vaccinat*"[tw] OR "vaccine*"[tw] OR "Vaccines"[Mesh]) AND  ("immunodeficien*"[tw] OR "immuno deficien*"[tw] OR "immunedeficien*"[tw] OR "immune deficien*"[tw] OR "immunologic deficiency syndromes"[tw] OR "PID"[tw] OR "CVID"[tw] OR "SCID"[tw] OR "hyper IgM syndrome*"[tw] OR "hyper-IgM syndrome*"[tw] OR "asplenia"[tw] OR "splenectomy"[tw] OR "aged"[tw] OR "elder*"[tw] OR "older adult*"[tw] OR "immunosenescence"[tw] OR "immunesenescence"[tw] OR "senescen*"[tw] OR "ageing"[tw] OR "aging"[tw] OR "inflammaging"[tw] OR "inflamm-aging"[tw] OR "preterm"[tw] OR "neonat*"[tw] OR "newborn"[tw] OR "premature"[tw] OR "baby"[tw] OR "babies"[tw] OR "age-associated"[tw] OR "infant"[tw] OR "pregnan*"[tw] OR "pregnant"[tw] OR "immunosuppression"[tw] OR "immunosuppress*"[tw] OR "lymphocyte depletion"[tw] OR "B-cell deplet*"[tw] OR "B cell deplet*"[tw] OR **"** immunosuppressive agents"[Mesh] OR "stem cell transplantation"[tw] OR "HSCT"[tw] OR "rituximab"[tw] OR "Rituxan"[tw] OR "Mabthera"[tw] OR "Rixathon"[tw] OR "Ruxience"[tw] OR "Truxima"[tw] OR "Blinatumomab"[tw] OR "Daratumumab"[tw] OR "Inotuzumab Ozogamicin"[tw] OR "Isatuximab"[tw] OR "Ocrelizumab"[tw] OR "Ofatumumab"[tw] OR "Obinutuzumab"[tw] OR "anti-CD20"[tw] OR "anti-CD19"[tw] OR "anti-CD38"[tw] OR "immunologic deficiency syndromes"[Mesh] OR "splenectomy"[Mesh] OR "aged"[Mesh] OR "immunosenescence"[Mesh] OR "aging"[Mesh] OR "infant"[Mesh] OR "immunosuppression"[Mesh] OR "stem cell transplantation"[Mesh] OR "rituximab"[Mesh] OR "Inotuzumab Ozogamicin"[Mesh]) AND "English" [Language] AND (("2000"[Date - Publication] : "3000"[Date - Publication]))

**Search strategy used for EMBASE database**

(exp B lymphocyte/ OR "B-Lymphocyte*".ti,ab. OR "B lymphocyte*".mp OR "B cell*".ti,ab. OR "B-cell*".ti,ab. OR "plasma cell*".ti,ab.) AND (exp "Vaccination"/ OR "vaccinat*".ti,ab. OR "vaccine*".ti,ab. OR exp vaccine/) AND  ("immunodeficien*".ti,ab. OR "immuno deficien*".ti,ab. OR "immunedeficien*".ti,ab. OR "immune deficien*".ti,ab. OR "immunologic deficiency syndromes".ti,ab. OR "PID".ti,ab. OR "CVID".ti,ab. OR "SCID".ti,ab. OR "hyper IgM syndrome*".ti,ab. OR "hyper-IgM syndrome*".ti,ab. OR "asplenia".ti,ab. OR "splenectomy".ti,ab. OR "aged".ti,ab. OR "elder*".ti,ab. OR "older adult*".ti,ab. OR "immunosenescence".ti,ab. OR "immunesenescence".ti,ab. OR "senescen*".ti,ab. OR "ageing".ti,ab. OR "aging".ti,ab. OR "inflammaging".ti,ab. OR "inflamm-aging".ti,ab. OR "preterm".ti,ab. OR "neonat*".ti,ab. OR "newborn".ti,ab. OR "premature".ti,ab. OR "baby".ti,ab. OR "babies".ti,ab. OR "age-associated".ti,ab. OR "infant".ti,ab. OR "pregnan*".ti,ab. OR "pregnant".ti,ab. OR "immunosuppression".ti,ab. OR "immunosuppress*".ti,ab. OR "lymphocyte depletion".ti,ab. OR "B-cell deplet*".ti,ab. OR "B cell deplet*".ti,ab. OR exp "immunosuppressive agent"/ OR "stem cell transplantation".ti,ab. OR "HSCT".ti,ab. OR "rituximab".ti,ab. OR "Rituxan".ti,ab. OR "Mabthera".ti,ab. OR "Rixathon".ti,ab. OR "Ruxience".ti,ab. OR "Truxima".ti,ab. OR "Blinatumomab".ti,ab. OR "Daratumumab".ti,ab. OR "Inotuzumab Ozogamicin".ti,ab. OR "Isatuximab".ti,ab. OR "Ocrelizumab".ti,ab. OR "Ofatumumab".ti,ab. OR "Obinutuzumab".ti,ab. OR "anti-CD20".ti,ab. OR "anti-CD19".ti,ab. OR "anti-CD38".ti,ab. OR exp immune deficiency/ OR exp "splenectomy"/ OR exp "aged"/ OR exp "immunosenescence"/ OR exp "aging"/ OR exp "infant"/ OR exp immunosuppressive treatment/ OR exp "stem cell transplantation"/ OR exp "rituximab"/ OR exp inotuzumab ozogamicin/) AND "English".lg. AND 2000:2021.(sa_year). NOT (conference OR conference abstract OR "conference review").pt.

**Search strategy used for Web of Science database***Results were restricted to language (English) and document type (Article).*

TS=("B-Lymphocyte*" OR "B cell*" OR "B-cell*" OR "B lymphocyte*" OR "plasma cell*") AND TS=("vaccinat*" OR "vaccine*") AND  TS=("immunodeficien*" OR "immuno deficien*" OR "immunedeficien*" OR "immune deficien*" OR "immunologic deficiency syndrome*" OR "PID" OR "CVID" OR "SCID" OR "hyper IgM syndrome*" OR "hyper-IgM syndrome*" OR "asplenia" OR "splenectomy" OR "aged" OR "elder*" OR "older adult*" OR "immunosenescence" OR "immunesenescence" OR "senescen*" OR "ageing" OR "aging" OR "inflammaging" OR "inflamm-aging" OR "preterm" OR "neonat*" OR "newborn" OR "premature" OR "baby" OR "babies" OR "age-associated" OR "infant*" OR "pregnan*" OR "pregnant" OR "immunosuppress*" OR "lymphocyte depletion" OR "B-cell deplet*" OR "B cell deplet*" OR "stem cell transplantation" OR "HSCT" OR "rituximab" OR "Rituxan" OR "Mabthera" OR "Rixathon" OR "Ruxience" OR "Truxima" OR "Blinatumomab" OR "Daratumumab" OR "Inotuzumab Ozogamicin" OR "Isatuximab" OR "Ocrelizumab" OR "Ofatumumab" OR "Obinutuzumab" OR "anti-CD20" OR "anti-CD19" OR "anti-CD38") AND PY=(2000-2020)

**Search strategy used for Academic Search Premier database***Results were restricted to language (English), source (Scholarly (Peer Reviewed) Journals) and publication date (20000101-20211231).*

("B-Lymphocyte*" OR "B cell*" OR "B-cell*" OR "B lymphocyte*" OR "plasma cell*") AND ("vaccinat*" OR "vaccine*") AND   ("immunodeficien*" OR "immuno deficien*" OR "immunedeficien*" OR "immune deficien*" OR "immunologic deficiency syndrome*" OR "PID" OR "CVID" OR "SCID" OR "hyper IgM syndrome*" OR "hyper-IgM syndrome*" OR "asplenia" OR "splenectomy" OR "aged" OR "elder*" OR "older adult*" OR "immunosenescence" OR "immunesenescence" OR "senescen*" OR "ageing" OR "aging" OR "inflammaging" OR "inflamm-aging" OR "preterm" OR "neonat*" OR "newborn" OR "premature" OR "baby" OR "babies" OR "age-associated" OR "infant*" OR "pregnan*" OR "pregnant" OR "immunosuppress*" OR "lymphocyte depletion" OR "B-cell deplet*" OR "B cell deplet*" OR "stem cell transplantation" OR "HSCT" OR "rituximab" OR "Rituxan" OR "Mabthera" OR "Rixathon" OR "Ruxience" OR "Truxima" OR "Blinatumomab" OR "Daratumumab" OR "Inotuzumab Ozogamicin" OR "Isatuximab" OR "Ocrelizumab" OR "Ofatumumab" OR "Obinutuzumab" OR "anti-CD20" OR "anti-CD19" OR "anti-CD38")

**Search strategy used for Cochrane database**

("B-Lymphocytes" OR "B-Lymphocyte*" OR "B cell*" OR "B-cell*" OR "B lymphocyte*" OR "B-lymphocyte*" OR "plasma cell*") AND ("Vaccination" OR "vaccinat*"OR "vaccine*" OR "Vaccines") AND  ("immunodeficien*" OR "immuno deficien*" OR "immunedeficien*" OR "immune deficien*" OR "immunologic deficiency syndromes" OR "PID" OR "CVID" OR "SCID" OR "hyper IgM syndrome*" OR "hyper-IgM syndrome*" OR "asplenia" OR "splenectomy" OR "aged" OR "elder*" OR "older adult*" OR "immunosenescence" OR "immunesenescence" OR "senescen*" OR "ageing" OR "aging" OR "inflammaging" OR "inflamm-aging" OR "preterm" OR "neonat*" OR "newborn" OR "premature" OR "baby" OR "babies" OR "age-associated" OR "infant" OR "pregnan*" OR "pregnant" OR "immunosuppression" OR "immunosuppress*" OR "lymphocyte depletion" OR "B-cell deplet*" OR "B cell deplet*" OR **"** immunosuppressive agents" OR "stem cell transplantation" OR "HSCT" OR "rituximab" OR "Rituxan" OR "Mabthera" OR "Rixathon" OR "Ruxience" OR "Truxima" OR "Blinatumomab" OR "Daratumumab" OR "Inotuzumab Ozogamicin" OR "Isatuximab" OR "Ocrelizumab" OR "Ofatumumab" OR "Obinutuzumab" OR "anti-CD20" OR "anti-CD19" OR "anti-CD38" OR "immunologic deficiency syndromes" OR "splenectomy" OR "aged" OR "immunosenescence" OR "aging" OR "infant" OR "immunosuppression" OR "stem cell transplantation" OR "rituximab" OR "Inotuzumab Ozogamicin"):ti,ab,kw AND la = english AND py=( 2000 OR 2001 OR 2002 OR 2003 OR 2004 OR 2005 OR 2006 OR 2007 OR 2008 OR 2009 OR 2010 OR 2011 OR 2012 OR 2013 OR 2014 OR 2015 OR 2016 OR 2017 OR 2018 OR 2019 OR 2020 OR 2021
