# Supplementary material for: B-Cell Immunophenotyping to Predict Vaccination Outcome in the Immunocompromised - A Systematic Review
Source: Front Immunol. 2021 Sep 7;12:690328. doi: 10.3389/fimmu.2021.690328 (PMC8452967; doi:10.3389/fimmu.2021.690328)
Supplement: Supplementary file 2 [file DataSheet_2.docx]

**Supplemental information- Supplemental Tables 1-7**

**Supplemental Table 1.** **Overview of the phenotypic descriptions used in the reviewed articles.** Only unique phenotypic descriptions are shown. Of note, several publications used overlapping phenotypic descriptions, but different population names. Therefore, some here-reported B-cell populations have high overlap in their phenotypic description. In cases where phenotypic descriptions were identical, both names were put in the same cell.

| **Regular B-cell populations** | **Phenotypic description used by reviewed articles** |
| --- | --- |
| Total B cells | CD19^+^  CD20^+^  CD19^+^CD20^+^  CD19^+^CD45^+^HLA-DR^+^ |
| B1 B cells | CD19^+^CD5^+^ |
| Transitional/Immature B cells | CD19^+^CD10^+^  CD20^+^CD10^+^  CD19^+^CD27^-^IgD^-^  CD19^+^CD24^hi^CD38^hi^  CD19^+^IgM^hi^CD38^hi^  CD19^+^CD21^int^IgM^hi^CD38^hi^  CD19^+^CD20^-^CD21^-^CD27^-^  CD19^+^CD20^+^CD27^+/-^IgD^+^CD38^+/-^  CD19^+^CD27^-^IgD^+^IgM^+/-^CD38^+^  CD19^+^CD20^+^CD10^+^CD23^+^CD5^+^CD38^+^ |
| Naive B cells/Mature Naive B cells | CD19^+^CD27^-^  CD19^+^CD27^-^IgD^+^  CD20^+^CD27^-^IgD^+^  CD19^+^CD27^-^IgM^+^  CD19^+^CD27^-^IgM^+^IgD^+^  CD19^+^CD27^-^CD21^+^CD38^+^  CD19^+^CD20^+^CD27^-^IgD^+^CD38^-^  CD19^+^CD27^-^IgG^-^IgA^-^  CD19^+^CD10^-^CD21^+^CD27^-^  CD19^+^CD10^-^CD20^+^CD21^+^CD27^-^  CD19^+^CD10^-^CD24^+^IgD^+^CD38^-^ |
| Memory B cells | CD19^+^CD27^+^  CD20^+^CD27^+^  CD19^+^CD20^+^CD27^+^  CD19^+^CD27^+^CD10^-^  CD20^+^CD27^+^CD10^-^  CD20^+^CD21^hi^CD27^+^CD10^-^ |
| Unswitched Memory B cells  (of note, high overlap with phenotypic description of IgM memory B cells between publications) | CD19^+^CD27^+^IgM^+^  CD19^+^CD27^+^IgM^hi^  CD19^+^CD27^+^IgM^hi^IgD^-^  CD20^+^CD27^+^IgM^+^IgD^+^  CD19^+^CD20^+^CD27^+^IgD^+^CD38^-^ |
| Marginal zone (like) B cells/Natural effector B cells | CD19^+^CD27^+^IgM^hi^IgD^+^  CD19^+^CD27^+^CD38^-^IgM^+^IgD^+^  CD19^+^CD27^+^IgM^+^IgD^+^ |
| IgM memory B cells/IgM only memory B cells | CD19^+^CD27^+^IgD^+^  CD19^+^CD27^+^IgM^+^  CD19^+^CD27^+^IgM^+^IgD^+^  CD19^+^CD27^+^IgM^hi^IgD^-^  CD19^+^CD27^+^IgM^hi^IgD^lo^CD21^hi^  CD19^+^CD10^-^CD27^+^CD21^hi^IgM^+^  CD19^+^CD27^+^IgD^+^IgG^-^IgA^-^ |
| IgD memory B cells | CD19^+^CD27^+^IgD^+^  CD19^+^CD27^+^CD38^lo^IgD^+^ |
| Switched Memory B cells | CD19^+^CD27^+^IgD^-^  CD20^+^CD27^+^IgD^-^  CD19^+^CD27^+^IgM^-^  CD19^+^CD27^+^IgM^-^IgD^-^  CD19^+^CD20^+^CD27^+^IgD^-^  CD19^+^CD27^+^CD38^lo^IgD^-^  CD19^+^CD20^+/-^CD27^+^IgD^-^CD38^-^  CD19^+^CD27^+^IgG^+^/IgA^+^  CD19^+^CD27^+^CD21^+^IgM^-^  CD19^+^CD27^+^CD38^+^IgM^-^IgD^‑^  CD19^+^CD10^-^CD27^+^CD21^hi^IgM^-^ |
| Resting memory B cells | CD19^+^CD10^-^CD20^+^CD21^+^CD27^+^  CD19^+^CD21^+^CD27^+^ |
| Activated memory B cells | CD19^+^CD10^-^CD20^+^CD21^-^CD27^+^  CD19^+^CD21^-^CD27^+^ |
| Activated (mature) B cells | CD20^+^CD21^lo^CD27^-^CD10^+^  CD19^+^CD10^-^CD21^-^  CD19^+^CD38^+^HLA-DR^+^ |
| Plasmablasts | CD19^+^CD27^hi^CD38^hi^CD45^+^HLA-DR^+^  CD19^+^CD10^-^CD20^-^CD27^hi^CD38^hi^  CD19^+^CD27^++^CD20^-^ (and Ag-specific)  CD19^+^CD27^+^CD38^+^  CD19^+^CD27^hi^CD38^hi^  CD19^lo^CD27^hi^CD38^hi^  CD20^lo^CD27^hi^CD38^hi^  CD19^+^CD27^+^CD38^+^CD24^-^  CD19^lo^CD21^int^CD38^hi^IgM^-(+)^  CD19^+^CD27^+^CD38^+^IgD^-^CD21^-^  CD20^lo^CD27^+^CD21^+^CD38^+^Ki67^-^  CD20^-^CD27^+^CD21^lo^CD10^-^Ki67^+^ |
| Antibody secreting cells | CD19^+^CD20^-^CD27^hi^CD38^hi^  CD19^+^CD20^lo^CD27^hi^CD38^hi^ |
| **Aberrant or immune senescence-related B-cell populations** |  |
| Atypical memory B cells | CD19^+^CD10^-^CD20^+^CD21^-^CD27^-^ |
| Age-associated B cells | CD19^+^CD21^-^T-bet^+^CD11c^+^CD27^+^ ‘memory’  CD19^+^CD21^-^T-bet^+^CD11c^+^CD27^-^ ‘atypical memory’ |
| Late/exhausted memory B cells/ double negative B cells | CD19^+^CD27^-^IgD^-^  CD19^+^CD27^-^CD21^lo^  CD19^+^CD20^+^CD27^-^IgD^-^CD38^-^  CD20^+^CD27^-^IgD^-^  CD19^+^CD27^-^IgD^-^ |
| Anergic B cells/CD21^lo^ B cells | CD19^+^CD21^lo^CD38^lo^  CD19^+^CD21^lo^CD38^lo^IgM^-^  CD19^+^CD21^-/lo^CD38^-^  CD19^+^CD27^-^CD21^lo^CD38^lo^ |
| Tissue-like (exhausted) memory B cells | CD20^+^CD10^-^CD21^lo^CD27^-^  CD19^+^CD21^-^CD27^-^IgD^-^CD38^+^  CD19^+^CD21^-^CD27^-^  CD19^+^CD20^+^CD21^-^CD27^-^  CD19^+^CD10^-^CD21^-^CD27^-^ |

In cases where ‘++’ was used to indicate high marker expression, this was replaced by ‘hi’ to make phenotypic descriptions more consistent.

**Supplemental Table 2.** **Overview of vaccines evaluated in reviewed studies.**

| **Overall vaccine group name** | **Commercial name(s)** | **Manufacturer(s)** | **Vaccine content (only the antigens are listed in this table)** |
| --- | --- | --- | --- |
| Mengingococcal vaccine- CRM carrier **(MenC-CRM)** | Menjugate® | Novartis vaccines and Diagnostics, GSK | *N. meningitidis* group C (strain c11) oligosaccharide (10µg), conjugated to CRM_197_ protein (12.5-25.0 µg) in 0.5 mL |
|  | | | |
| Pneumococcal conjugate vaccines **(PCV)** | Prevnar 7®/ Prevenar 7® | Pfizer, Wyeth | Polysaccharides of 7 *S. pneumoniae* serotypes (4, 6B, 9V, 14, 18C, 19F, and 23F), conjugated with diphtheria proteins (CRM_197_); 2mg of each (except for 6B, of which 4mg is present, 20mg of CRM_197_ |
|  | Prevnar 13®/ Prevenar 13® | Pfizer, Wyeth | Polysaccharides of 13 *S. pneumoniae* serotypes (1, 3, 4, 5, 6A, 6B, 7F, 9V, 14, 18C, 19A, 19F, and 23F), conjugated with diphtheria proteins (CRM_197_); 2.2mg of each (except for 6B, of which 4.4mg is present), 34mg of CRM_197_ |
| Pneumococcal polysaccharide vaccines **(PPV)** | Pneumovax  Pneumovax 23®,  Pneumo 23®,  Pneumovax II,  Pnu-Immune® | Merck, MSD,  Merck, MSD, Sanofi Pasteur  Aventis Pasteur  Aventis Pasteur, Sanofi Pasteur MSD  Lederle Laboratories, Wyeth | Polysaccharides of 23 *S. pneumoniae* serotypes (1, 2, 3, 4, 5, 6B, 7F, 8, 9N, 9V, 10A, 11A, 12F, 14, 15B, 17F, 18C, 19A, 19F, 20, 22F, 23F, and 33F), 25mg of each |
|  | | | |
| Influenza vaccines | Pandemrix® | GSK | Monovalent: A/California/7/2009 (H1N1)-like virus; 3.75mg HA per 0.5ml |
|  | Fluarix®,  TIV 2010-2011,  TIV 2011-2012 Fluzone®, TIV 2011-2012 Fluvirin®, TIV 2010-2011 Vaxigrip®, TIV 2010-2011 | GSK  No manufacturer indicated  No manufacturer indicated  Sanofi Pasteur Novartis Sanofi Pasteur | Trivalent, (1) A/ California/7/2009 (H1N1), (2) A/Perth/16/2009 (H3N2), (3) B/ Brisbane/60/2008, 15mg HA per 0.5ml |
|  | Flucelvax®, TIV 2014-2015 | Novartis | Trivalent, (1) A/Brisbane/10/2010 (H1N1, A/California/7/2009-like virus), (2) NYMC X-223A (H3N2, A/Victoria/361/2011-like virus), (3) B/Massachusetts/2/2012 |
|  | Fluvirin®, pH1N1 2009, TIV 2013-2014, QIV 2015-2016, | Novartis | Monovalent: A/California/7/2009 (H1N1)-like virus; 15mg HA per 0.5ml  Trivalent, in 2013-2014 season: (1) A/Christchurch/16/2010 (H1N1); (2) A/Texas/50/2012 (H3N2); (3) B subtype virus, B/Massachusetts/2/2012  Quadrivalent, in 2015-2016 season: (1) A/Christchurch/16/2010, (2) NIB74 (H1N1, A/California/7/2009 pdm09-like virus), (3) NIB-88 (H3N2 A/Switzerland/9715293/2013-like virus), (4) B/Phuket/3073/2013. |
|  | Agriflu® | Novartis | Trivalent: (1)A/H1N1 (2007), (2) A/H3N2 (2007), (3) B/Brisbane |
|  | Intanza®, TIV 2010-2011 | Sanofi Pasteur | Trivalent: (1) A/California/7/2009 (H1N1), (2) A/Perth/16/2009 (H3N2), (3) B/Brisbane/60/2008 (18 µg per antigen per dose) |
|  | Vaxigrip®, TIV 2012-2013, pH1N1 2009 | Sanofi Pasteur | Monovalent: A/California/7/2009 (H1N1)-like virus; 15mg HA per 0.5ml  Trivalent: (1) A/California/7/ 2009 (H1N1) pdm09-like virus, (2) A/Victoria/361/2011 (H3N2)-like virus, (3) B/Wisconsin/1/2010-like virus; 15mg HA per 0.5ml |
|  | Vaxigrip®, TIV 2005-2006 | SBL Vaccin AB | Trivalent, (1) A/New Caledonia/20/99 (H1N1), (2) A/California/7/2004 (H3N2), (3) B/Shanghai/361/2002; 15mg HA per 0.5ml |
|  | Fluvax®, TIV 2015-2016 | bioCSL | Trivalent: (1) A/California/7/2009-like virus (H1N1), (2) A/Switzerland/9715293/2013 (H3N2)-like virus and (3) B/Phuket/3073/2013-like virus; 15mg HA per 0.5ml |
|  | Influvac®, TIV 2007-2008 | Solvay | Trivalent, (1) A/Wisconsin/67/2005-like virus (H3N2), (2) A/Solomon Islands/3/2006-like virus (H1 N1), (3) B/Malaysia/2506/2004-like virus, 15 mg HA per 0.5ml |
|  | Inflexal V®, TIV 2008-2009 | Crucell | Trivalent, (1) A/ Brisbane/10/2007 (H3N2), (2) A/Brisbane/59/2007 (H1N1), (3) B/Florida/4/ 2006, 15 mg HA per dose |
|  | Focetria® | Novartis | Monovalent MF-59–adjuvanted vaccine: A/California/7/ 2009(H1N1) pdm09 |
|  | Fluad® | Novartis | Trivalent, (1) A/Brisbane/10/2007 (H3N2), (2) B/Brisbane/60/2008 (B), (3) A/Brisbane/59/2007 (H1N1), 15 mg HA per 0.5ml |
|  | TIV 2006-2007 | Sanofi Pasteur | Trivalent, (1) A/New Caledonia/20/99 (H1N1), (2) A/Wisconsin/67/2005 (H3N2), and (3) B/Malaysia/2506/2004, 15 mg HA per dose |
|  | TIV 2009-2010 | No manufacturer indicated | Trivalent, A/Brisbane/ 59/2007 (H1N1)-like virus; A/Brisbane/10/2007 (H3N2)-like virus; B/Brisbane/60/2008-like virus. |
|  | TIV 2014-2015 | No manufacturer indicated | Trivalent, (1) A/California/7/2009, (2) A/Texas/50/2012, (3) B/Mass/12 |
|  | QIV 2015-2016 | No manufacturer indicated | Quadrivalent, (1) A/California/7/2009, (2) A/Switzerland/9715293/2013, (3) B/Phuket/13, (4) B/Brisb/08 |
|  | QIV 2016-2017 | No manufacturer indicated | Quadrivalent, (1) A/California/7/2009, (2) A/Hong kong/4801/2014, (3) B/Phuket/13, (4) B/Brisb/08 |
|  | | | |
| Hepatitis vaccines | Engerix-B® | GSK | Hepatitis B surface antigen (HBsAg) recombinant (yeast) vaccine; typically 10 or 20mg per 0.5ml dose |
|  | Recombivax-HB® | MSD | Hepatitis B surface antigen (HBsAg) synthetic; 10-20mg per 1ml dose |
|  | Twinrix® | GSK | Hepatitis A (inactivated) and hepatitis B (rDNA) (HAB) vaccine; 720 ELISA Units hepatitis A virus (inactivated) and 20mg hepatitis B surface antigen per 1ml dose |
|  | Havrix® | GSK | Hepatitis A Vaccine 1440 Units per 1ml dose |
|  | | | |
| Combination vaccines | Pentavac® | Sanofi Pasteur | Diphtheria (≥30 IU), tetanus toxoids (≥40 IU), acellular pertussis adsorbed (25 ug of pertussis toxoid, 25 ug of filamentous haemagglutinin (FHA)), inactivated poliovirus (40 U type 1, 8 U type 2, 32 U type 3) and *H. influenzae type b* vaccine (10 ug); 0.5 ml dose |
|  | Tetagrip® | Sanofi Pasteur | Tetanus toxoids and trivalent influenza vaccine; (1) A/New Caledonia/20/99 (H1N1), (2) A/Wisconsin/67/2005 (H3N2), and (3) B/Malaysia/2506/2004, 15 mg HA per 0.5 ml dose |
|  | Decavac® | Sanofi Pasteur | Tetanus (5 Lf) and diphtheria (2 Lf) per 0.5 mL dose |
|  | Duplex® | SBL | Diphtheria (30 Lf/mL) and tetanus toxoids (7.5 Lf/mL), 0.25 ml dose |
|  | Boostrix ® | GSK | Tetanus (≥5 Lf), diphtheria (≥2.5 Lf), acellular pertussis (8 ug pertussis toxoid, 8 ug FHA, 2.5 ug pertactin) adsorbed per 0.5 ml dose |
|  | | | |
| Tick-borne encephalitis vaccines | FSME Immun® | Pfizer | Inactivated tick-borne encephalitis virus strain Neudörfl vaccine; 2.4 ug per 0.5 ml dose |
|  | | | |
| Hemophilus influenzae type vaccines | Act-Hib® | Sanofi Pasteur | *H. influenzae type b* vaccine conjugated to 18-30 ug tetanus toxoid, 10 ug per 0.5 ml dose |
|  | | | |
| Immunocyanin vaccines | Immucothel® | biosyn | Immunocyanin (keyhole limpet hemocyanin) vaccine, 1 mg in 1 ml dose |
|  | | | |
| HPV vaccines | Cervarix® | GSK | Bivalent, HPV16 (20 ug), HPV18 (20 ug) per 0.5 ml dose |

abbreviations: TIV, trivalent influenza vaccine; QIV, quadrivalent influenza vaccine; HPV, human papilloma virus

**Supplemental Table 3.** **Overview of vaccination studies in elderly.**

| **Authors** | **Year** | **Cohort** | **Vaccine** | **Baseline assays** | **Follow-up, incl. timing of measurement** | **Risk of bias** |
| --- | --- | --- | --- | --- | --- | --- |
| Ademokun et al. | 2011 | 27 elderly, aged 65-89 years  39 adults, aged 18-49 years | Influvac (Solvay) +  PPV (Pneumovax II®; Sanofi Pasteur MSD) | ELISA, spectratyping | ELISA, spectratyping  d7, d28 | low |
| Carson et al. | 2000 | 29 elderly, aged 75-103 years  21 adults, aged 25-35 years | PPV (Pnu-Immune; Lederle Laboratories)  +  Alum-absorbed ultrafine TT+DT vaccine (Wyeth Laboratories Inc.) | FC, nephelometry, ELISA, Antibody avidity, MNC stimulation | ELISA, antibody avidity, MNC stimulation  d28 | moderate |
| Kolibab et al. ^a^ | 2005 | 20 elderly, aged >65 years  20 adults, aged <30 years | PPV (Pneumovax®; Merck & Co. Inc.) | Baseline data reported in Kolibab et al., 2005b | repertoire analysis Ag-specific B cells  w6 | low |
| Kolibab et al. ^b^ | 2005 | 20 elderly, aged >65 years  20 adults, aged <30 years | PPV (Pneumovax®; Merck & Co. Inc.) | CBC, Blood Chemistry profile, ELISA, IgG antibody avidity, OPK assay | ELISA, IgG antibody avidity, OPK assay  w6 | moderate |
| Leggat et al. | 2013 | 14 elderly, aged 64-88 years  18 adults, aged 18-30 years | PPV (Pneumovax 23®, Merck) | FC, ELISA, OPK assay | FC  d7  ELISA, OPK assay  d28 | moderate |
| Shi et al. | 2005 | 65 elderly, aged 65-99 years  65 adults, aged 21-64 years | PPV (Pneumovax®, manufacturer not specified) | FC, latex agglutination test, ELISA | FC, latex agglutination test, ELISA  w4 | low |
| Abreu et al. | 2020 | 35 elderly, aged 65-85 years  24 adults, aged 18-34 years | TIV/QIV: 2014, 2015, 2016  (manufacturer not specified) | FC, HAI assay, ELISA | FC, HAI assay, ELISA, isotype-specific antibody fractionation  d21/28 | moderate |
| Camous et al. | 2018 | 22 elderly, aged 65-84 years  29 adults, aged 23-33 years | Vaxigrip® (Sanofi Pasteur) | FC, HAI assay, microneutralization assay | FC  d2, d7, d28  HAI assay, microneutralization assay  d28 | low |
| Frasca et al. | 2011 | 9 elderly, aged 65-75 years  34 adults, aged 20-64 years | pH1N1 2009 (Novartis, monovalent) /  pH1N1 2009 (Sanofi Pasteur monovalent)  + Seasonal influenza vaccine of current and past 3 seasons (not specified) | FC, HAI assay, ELISA, qPCR, RT PCR | FC  d7, d28 (w4/6)  HAI assay, ELISA, qPCR, RT-PCR  d28 (w4/6) | low |
| Frasca et al. | 2017 | 6 elderly, aged > 65 years  6 adults, aged 25-55 years | TIV 2011/2012 (manufacturer not specified) | FC, FACS, qPCR, HAI assay, WB | FC, FACS, qPCR, HAI assay, WB  d7, d28 (w4/6) | low |
| Kannan et al. | 2015 | 35 elderly, aged 66-88 years  28 adults, aged 30-40 years | TIV 2012/2013 (manufacturer not specified) | FC, microneutralization assay, ELISA | FC, microneutralization assay, ELISA  d7, d14 | moderate |
| Kurupati et al. | 2013 | 30 elderly, aged 65-87 years  15 adults, aged 30-40 | TIV 2011/2012 (manufacturer not specified) | FC, microneutralization assay, ELISpot, ELISA | FC, microneutralization assay, ELISpot  d0, d7, d10, d14, d28, d60  ELISA  d10, d28 | low |
| Nipper et al. | 2018 | 151 elderly, aged 64-95 years  55 adults, aged 22-45 years | TIV 2010/2011 or TIV 2011/2012 (manufacturer not specified) | HAI assay | FC, HAI assay  m2-4 | serious |

Abbreviations: CRM, cross-reactive material; TT, tetanus toxoid; DT, diphtheria toxoid; TIV, trivalent influenza vaccine; QIV, quadrivalent influenza vaccine; IIV, inactivated influenza vaccine; Hib, *H. influenzae* type b; RT PCR, reverse transcriptase polymerase chain reaction; ELISA, enzyme-linked immunosorbent assay; ELISpot, enzyme-linked immune absorbent spot; (r)SBA, (rabbit) serum bactericidal activity; FC, flow cytometry; OPK, opsonophagocytic killing; MNC, mononuclear cells; CBC , complete blood count; Ig, immunoglobulin; HAI, hemagglutination inhibition; FACS, fluorescence activated cell sorting; WB, Western Blot; d, days; w, weeks; m, months.

**Supplemental Table 4.** **Overview of vaccination studies in immunodeficient patients.**

| **Authors** | **Year** | **Cohort** | **Treatment** | **Vaccine** | **Baseline assays** | **Follow-up, incl. timing of measurement** | **Risk of Bias** |
| --- | --- | --- | --- | --- | --- | --- | --- |
| Cavaliere et al. | 2013 | 125 CVID patients  20 healthy controls | SCIG or IVIG (125) | PPV (Pneumovax®, Merck) | FC, ELISA | ELISA  d0, d28, d360 | low |
| Gardulf et al. | 2018 | 48 CVID patients (initially 57 recruited) | SCIG (47)  IVIG (1) | Pandemrix (GSK) | FC (at diagnosis), HAI assay, ELISA*, PCR* | HAI assay  d0, m1, m3 | moderate |
| Goldacker et al. | 2007 | 21 CVID patients | Ig substitution therapy (21) | routine vaccines: tetanus and diphtheria, Hib, PPV, hepatitis A/B (Twinrix®, GSK) | FC, PBMC stimulation, analysis of total IgM, IgA, IgG levels, ELISA | ELISA  in regular intervals for up to 1y | low |
| Ko et al. | 2005 | 53 CVID patients  30 healthy controls | Ig substitution therapy (part of the donors) | PPV (Pneumovax®, Merck) | FC, ELISA | ELISA  d0, w4-6 | low |
| Pulvirenti et al. | 2020 | 74 CVID patients  20 healthy controls | SCIG or IVIG (all) | PPV (Pneumovax®, Merck) | FC, ELISA | ELISA  d0, w4, m36 ± 6 | low |
| Rezaei et al. | 2005 | 12 (pediatric) CVID patients | NI | meningococcal polysaccharide vaccine (manufacturer not specified) | FC, SBA | SBA  d0, w3 | low |
| Sharifi et al. | 2018 | 16 CVID patients  16 healthy controls | NI | PPV (Pneumovax 23, manufacturer not specified) | FC, ELISA, PBMC stimulation*, RT-PCR* | ELISA  NI | low |
| Yazdani et al.^a^ | 2017 | 10 CVID patients  10 healthy controls | IVIG | PPV (Pneumo 23®, Aventis, Pasteur) | FC, B-cell stimulation, RT-PCR, ELISA | ELISA  d0, d21 | low |
| Yazdani et al.^b^ | 2017 | 30 CVID patients  30 healthy controls | NI | PPV (Pneumo 23®, Aventis, Pasteur) | FC, ELISA | ELISA  d0, d21 | low |
| Abudulai et al. | 2016 | 50 HIV patients  20 healthy controls | ART (30) | PPV (Pneumovax®, Merck) | FC, microsphere-based FC assay, analysis of Ig subclasses and serum free light chains | microsphere-based FC assay, ELISpot  d0, d7, d28 | moderate |
| Cagigi et al. | 2013 | 54 HIV patients  47 healthy controls | ART (37) | Pandemrix® (GSK) | HAI assay, PBMC stimulation, RT-qPCR, FC | HAI assay  d0, m1, m3, m6 | moderate |
| Cagigi et al. | 2014 | 59 HIV patients,  19 controls | ART (59) | Split Virion VAXIGRIP (Sanofi Pasteur) | FC, ELISA | ELISA  d0, d21 | low |
| Chang et al. | 2000 | 12 HIV patients  10 healthy controls | ART (6) | PPV (Pneumovax®, MSD) | PCR, PCR ELISA, ELISA | PCR, PCR ELISA, ELISA  d0, d7, d28 | low |
| Curtis et al. | 2015 | 90 HIV patients (children and youth) | NI | Fluvirin (Novartis) | FC, ELISA, HAI assay, FluoroSpot | FC, ELISA, HAI assay, FluoroSpot  d0, d21-28, d10-14 post dose 2, w28 post dose 1 | moderate |
| Eisen et al. | 2016 | 93 HIV patients  51 healthy controls | NI | PPV (Pneumovax 23®, manufacturer not specified) | FC, flow-based multiplex assay, OPK assay | flow-based multiplex assay, OPK assay  d0, d28, y1 | moderate |
| Farmaki et al. | 2018 | 40 HIV patients | ART (40) | PCV (Prevenar 13, Pfizer) and PPV (Pneumovax 23, MSD) after 12m | FC, ELISA | FC, ELISA  d0, m1 (after each vaccine) | low |
| Hart et al. | 2007 | 84 HIV patients  28 CVID patients  8 splenectomized patients  83 healthy controls | ART (55)  Ig replacement therapy (25) | TT (Aventis Pasteur) (14 patients) or PPV (Pneumovax II, Aventis Pasteur) (19 patients) | FC, ELISA | ELISA  d0, w4, m3-6 | moderate |
| Johannesson et al. | 2012 | 95 HIV patients | ART (62)  impaired ART responders (13) | PCV (Prevnar, Wyeth) 2 doses 3m apart with or without CPG7909 | FC, ELISA | ELISA  d0, m3, m4, m9 | moderate |
| Luo et al. | 2016 | 26 HIV patients  16 healthy controls | ART (26) | Fluvirin (Novartis) | FC, ELISpot, HAI assay, ELISA, microneutralization assay, qPCR | FC, ELISpot, HAI assay, ELISA, microneutralization assay  d0, d7-10, d14-21 | low |
| Milagres et al. | 2018 | 17 (pediatric) HIV patients  12 healthy controls | ART | MenC-CRM (Novartis; C Polysaccharide/CRM197), in patients, booster at y1 | SBA, FC, ELISA, turbidimetry | SBA, FC  d0, m1-2, m10-12 (booster), m1-2 (after booster) (controls d0, m1-2 only) | low |
| Pallikkuth et al.^a^ | 2011 | 17 HIV patients  8 healthy controls | ART (17) | A/California/ 07/2009 H1N1 vaccine (Novartis Vaccines and Diagnostics Ltd) | FC, ELISpot, ELISA | FC, ELISpot, ELISA  d0, d7, d28 | low |
| Pallikkuth et al.^b^ | 2011 | 16 HIV patients  8 healthy controls | ART (16) | A/California/ 07/2009 H1N1 vaccine (Novartis Vaccines and Diagnostics Ltd) | FC, ELISpot, ELISA, HAI assay | FC, ELISpot, ELISA, HAI assay  d0, d7, d28 | low |
| Paris et al. | 2017 | 20 HIV patients | ART (20) | 20 µg Engerix-B® or 10 µg Recombivax-HB® (0-, 1-, 6-month schedule) (manufacturer not specified) | FC, ELISA, Luminex assay | FC, ELISA, Luminex assay  d0, within 12m after the 3^rd^ dose | low |
| Parmigiani et al. | 2013 | 16 HIV patients  12 healthy controls | ART (16) | Fluarix® (GSK) | HAI assay, Luminex assay, ELISA, FC, cell co-cultures | HAI assay, ELISA, FC  t0, w4 | low |
| Rinaldi et al. | 2017 | 64 HIV patients  60 healthy controls | ART (64) | TIV, seasonal influenza vaccination, 2013/2014, 2014/2015 and 2015/2016 (manufacturer not specified) | HAI assay, FC, ELISpot | HAI assay, ELISpot  d0, d7, d21 | low |
| Tsachouridou et al. | 2015 | 66 HIV patients  60 healthy controls | HAART (35) | PPV (Pneumovax 23®, Merck and Co., Inc.) | FC, ELISA | ELISA  d0, w4, w48 | low |
| Van Epps et al. | 2014 | 46 HIV patients  30 healthy controls | ART (part) | TT (Aventis Pasteur or Wyeth-Ayerst), hepA vaccine (Smith-Kline Beecham) | FC, ELISA | FC, ELISA  d0, w192 | moderate |
| Weinberg et al. | 2009 | 152 (pediatric) HIV patients | NI | Havrix (GSK), two doses 24 weeks apart | ELISA, lymphocyte proliferation assay, Luminex, FC | ELISA, lymphocyte proliferation assay, Luminex, FC  d0, w32 (some analysis every 8w) | low |
| Wheatley et al. | 2016 | 26 HIV patients  30 healthy controls | ART (26) | 2015 IIV3 (Fluvax, bioCSL) | FC, HAI assay | FC, HAI assay  d0, w4 | low |
| Giesecke et al. | 2014 | 6 splenectomized and 3 tonsillectomized patients, 12 healthy controls (additional tissues from non-vaccinated donors) | - | TT/DT (Sanofi Pasteur MSD GmbH) | FC, ELISA | FC, ELISA  d0, d7, d14 | low |
| Papadatou et al. | 2014 | 39 patients with β-thalassemia and asplenia | - | PCV13 (previous vaccinations: PCV13, 1-4 doses of PPV23) (manufacturer not specified) | ELISpot, ELISA | ELISpot, ELISA  d0, d7, d28 | low |
| Rosado et al. | 2013 | 57 asplenic adults, 21 asplenic children, 47 healthy adults, 19 healthy children | - | PPV or PCV7 (manufacturer not specified), before and/or after splenectomy | NI | ELISpot*, ELISA*, FC*, IHC*, cell culture*  NI | moderate |
| Wasserstrom et al. | 2008 | 26 splenectomized patients  12 healthy controls | - | PPV (Pneumovax 23, Merck) | ELISA, FC | ELISA  d0, w4-7 | low |

Abbreviations: CVID, common variable immunodeficiency; HIV, human immunodeficiency virus; NI, not indicated; PPV, pneumococcal polysaccharide vaccine; PCV, pneumococcal conjugate vaccine; TT, tetanus toxoid; DT, diphtheria toxoid; hep, hepatitis; TIV, trivalent influenza vaccine; SCIG, subcutaneous Ig; IVIG, intravenous Ig; Hib, *H. influenzae* type b; RT (q)PCR, real time (quantitative) polymerase chain reaction; HAI, hemagglutination inhibition; SBA, serum bactericidal antibody; ART, Antiretroviral Therapy; HAART, Highly Active Antiretroviral Therapy; ELISA, enzyme-linked immunosorbent assay; ELISpot, enzyme-linked immune absorbent spot; OPK, opsonophagocytic killing; IHC, immunohistochemistry; d, day; w, week; m, month; y, year; *, not specified whether analysis performed at baseline; follow up or both.

| Name |  |  | | |  |  | | |
| --- | --- | --- | --- | --- | --- | --- | --- | --- |
| London | group A | **no** production of **IgM or IgG** upon stimulation *in vitro* | | | | | | |
|  | group B | production of **IgM**, but **not IgG** upon stimulation *in vitro* | | | | | | |
|  | group C | production of **IgM** and **IgG** upon stimulation *in vitro* | | | | | | |
| Freiburg* | group I | **class-switched MBCs** (CD27^+^IgD^-^IgM^-^) **<0.4%** of PBLs | | | a | **immature CD21^-^** B cells **>20%** of B cells | | |
|  |  |  |  |  | b | **immature CD21^-^** B cells **<20%** of B cells | | |
|  | group II | **class-switched MBCs** (CD27^+^IgD^-^IgM^-^) **>0.4%** of PBLs | | |  |  | | |
|  |  |  |  |  |  |  | | |
| Paris | MB0 | total (CD27^+^) **MBCs ≤11%** of PBLs | | | | | | |
|  | MB1 | total (CD27^+^) **MBCs >11%** of PBLs; **class-switched MBCs** (CD27^+^IgD^-^) **≤8%** of B cells | | | | | | |
|  | MB2 | total (CD27^+^) **MBCs >11%** of PBLs; **class-switched MBCs** (CD27^+^IgD^-^) **>8%** of B cells | | | | | | |
| EUROclass | B^-^ | **B cells** (CD19^+^) **<1%** PBLs |  | | | | | |
|  | B^+^ | **B cells** (CD19^+^) **>1%** PBLs | smB^-^ | **class-switched** (CD27^+^IgD^-^IgM^-^) **MBCs ≤2%** of B cells | | | TR^hi^ | transitional (CD38^++^IgM^high^ ) ≥9% of B cells |
|  |  |  |  |  |  |  | TR^norm^ | transitional (CD38^++^IgM^high^ ) <9% of B cells |
|  |  |  |  |  |  |  | 21^low^ | ≥10% CD21^low^ B cells |
|  |  |  |  |  |  |  | 21^norm^ | <10% CD21^low^ B cells |
|  |  |  | smB^+^ | **class-switched** (CD27^+^IgD^-^IgM^-^) **MBCs>2**% of B cells | | | 21^low^ | ≥10% CD21^low^ B cells |
|  |  |  |  |  |  |  | 21^norm^ | <10% CD21^low^ B cells |
| B-cell pattern | pattern 1 | B-cell production and germinal center defect | | | | | | |
|  | pattern 2 | early peripheral B-cell maturation or survival defect | | | | | | |
|  | pattern 3 | B-cell activation and proliferation defect | | | | | | |
|  | pattern 4 | germinal center defect | | | | | | |
|  | pattern 5 | post germinal center defect | | | | | | |

**Supplemental Table 5. Classifications of CVID patients**

*****, includes only CVID patients with peripheral B-cell numbers above 1% of peripheral blood lymphocytes (PBL)

**Supplemental Table 6. Classification of vaccination responders in CVID**

| Study | vaccine | Responders | London | Freiburg | Paris | EUROClass | B-cell pattern |
| --- | --- | --- | --- | --- | --- | --- | --- |
| Cavaliere et al. | PPV | 10/125 (IgM and IgA)  25/125 (IgM)  2/125 (IgA) | - | - | - | - | - |
| Gardulf  et al. | influenza | 8/48 | - | Ia:2  Ib:5  II:1 | MB0:1  MB1:6  MB2:1 | smB^+^21^low^:1  smB^-^21^low^ TR^hi^ :1  smB^-^21^norm^ TR^norm^ :6 | pattern 1: 2  pattern 3: 1  pattern 4: 4  pattern 5:1 |
| Goldacker et al | PPV  HepA  HepB  Tetanus  Diph  Hib | 3/15 (IgM)  3/17 (IgG)  1/17  7/17  1/13  2/14  6/18 | A:1  B:1  C:1  A:1  C:2  C:1  A:1  B:3  C:3  C:1  B:1  C:1  B:2  C:4 | Ib:2  II:1  Ib:1  II:2  II:1  Ia:1  Ib:4  II:2  II:1  Ib:1  II:2  Ib:3  II:3 | MB1:3  MB1:2  MB2:1  MB2:1  MB0:3  MB1:2  MB1:2  MB2:1  MB1:1  MB2:1  MB0:1  MB1:3  MB2:2 | - | - |
| Ko  et al. | PPV | - | - | - | - | - | - |
| Pulvirenti et al. | PPV | 14/76* | - | Ib:27%  II:73% | smB+:  100% | - | - |
| Rezaei  et al. | meningococcal  polysaccharide vaccine | 7/12 |  | Ia:2  Ib:3  II:2 |  |  |  |
| Sharifi  et al. | PPV | - | - | - | - | - | - |
| Yazdani  et al.^a^ | PPV | 2/8 | - | - | - | - | - |
| Yazdani  et al.^b^ | PPV | 3/25 |  | Ib:1  II:1 | MB0:2  MB1:1 | smB^-^21^low^ smB^-^Tr^norm^:1  smB^-^21^norm^ smB^-^Tr^norm^:1  smB^+^21^norm^:1 | pattern 3:2  pattern 4:1 |

*****, either 14 or 16 indicated in different sections of the manuscript, due to this inconsistency, number of donors belonging to different classifications expressed as % as it was done in the manuscript

**Supplemental Table 7. Overview of vaccination studies in immunosuppressed patients.**

| **Authors** | **Year** | **Cohort** | **Vaccine** | **Timing** | **Baseline assays** | **Follow-up, incl. timing of measurement** | **Risk of bias** |
| --- | --- | --- | --- | --- | --- | --- | --- |
| Avetisayan et al. | 2008 | 14 post-alloHCT patients Indication: malignancy Immunosuppression: heterogeneous 18 healthy controls | Vaxigrip® (SBL Vaccin AB) | Variable | ELISpot, HAI assay | w4, ELISpot and HAI assay. | moderate |
| Roll et al. | 2012 | 36 post-alloHCT patients Indication: heterogeneous Immunosuppression: heterogeneous | Pandemrix® (GSK) | NI | FC, HAI assay | w4-8, HAI assay. | moderate |
| Buckley et al. | 2013 | SCID patients post-alloHCT  61 on IVIG 64 off IVIG Immunosuppression: none  777 healthy controls | Tetanus, diphtheria vaccine (not specified) Neoantigen phiX174 (0.02 ml/kg intravenously) | NI | FC | Tanned red cell hemagglutination or ELISA, BCR spectratyping. | moderate |
| Hoshina et al. | 2016 | 8 post-HCT patients Indication: heterogeneous Immunosuppression: none 9 patients on immunosuppression Indication: heterogeneous Immunosuppression: heterogeneous 14 healthy controls | PCV (Prevnar 7®; Pfizer) | NI | FC, OPK assay, ELISA | w4-6. ELISA and multiplexed OPK assay. | moderate |
| Harrison et al | 2020 | 19 post-alloHCT patients  Indication: heterogeneous Immunosuppression: heterogeneous 15 healthy controls | FSME Immun® (Pfizer) | m0, w4, m9-12 | FC, neutralization assay | w4, Neutralization assay, ELISA. | moderate |
| Winkler et al. | 2020 | 27 post-alloHCT patients  Indication: heterogeneous Immunosuppression: heterogeneous 13 healthy controls | Pentavac® (Sanofi Pasteur) PCV (Prevenar 13®; Wyeth) | NI | FC, ELISA, ELISpot | w1, w4, w8, w26, w52. ELISA, ELISpot. | moderate |
| Puissant-Lubrano et al. | 2010 | Post-kidney Tx patients 26 on heterogeneous immunosuppression 13 on immunosuppression + rituximab 30 healthy controls | Tetagrip® (Sanofi Pasteur) | NI | FC, nephelometry, ELISA | m1, ELISA, FC. | moderate |
| Bedognetti et al. | 2011 | 31 NHL patients on heterogeneous immunosuppression + rituximab 34 healthy controls | Inflexal V® (Crucell) | Median m29 post-treatment | FC, HAI assay | w4, FC, HAI assay. | moderate |
| Pescovitz et al. | 2011 | Diabetes type I patients 46 on rituximab 29 on placebo | Decavac® (Sanofi Pasteur) Havrix® (GSK)  Subset: neoantigen phiX174 (University of Washington) | m12 after treatment  w6, w12, w52 and w58 | FC, ELISA | w4, ELISA for DT and HepA. PhiX194: w1, w2 and w4 after each immunization: phage neutralization assay. | some concerns |
| Bedognetti et al. | 2012 | 14 NHL patients on rituximab in addition to relatively homogeneous immunosuppression 21 healthy controls | Focetria® (Novartis; 2 doses) followed at d28 by Fluad® (Novartis) | NI | FC, HAI assay | w4, HAI assay. | moderate |
| Eisenberg et al. | 2013 | 25 heterogeneous auto-immune disease patients on rituximab + heterogeneous immunosuppression 15 healthy controls | TIV (2006–2007, 2007–2008, 2008–2009, 2009–2010) (manufacturer not specified) | m7-9 after treatment | FC, HAI assay, ELISpot | m2, m6, HAI assay, ELISpot. | moderate |
| Nazi et al. | 2013 | ITP patients 17 on rituximab  7 on placebo | PPV (Pneumovax 23®; Merck) Act-Hib® (Sanofi Pasteur) | m6 after rituximab or placebo | FC, ELISA, SBA (anti-Hib) | w1, w4 and m6, ELISA, SBA (anti-Hib), FC | some concerns |
| Cho et al. | 2017 | 23 pemphigus patients on rituximab 28 healthy controls | Flucelvax® (Novartis) or Fluvirin® (Novartis) | NI | FC, ELISpot, HAI assay | d7, ELISpot. d28, ELISpot, HAI assay, MN assay, PCR. | moderate |
| Ek et al. | 2005 | 31 ALL patients on NOPHO chemotherapy 40 healthy controls | Duplex® (SBL) Act-Hib® (Sanofi Pasteur) | m1 or m6 post-treatment | FC | d7, ELISpot.  d21, ELISA. | moderate |
| Chu et al. | 2013 | Ovarian cancer patients  13 on DC vaccine + chemotherapy 18 on heterogeneous chemotherapy 21 healthy controls | Seasonal inactivated trivalent influenza vaccine (manufacturer not specified) | NI | FC, HAI assay | m3, m4, m9, m12, HAI assay, ELISpot. | low |
| De Lavallade et al. | 2013 | 51 CP-CML patients on TKI 24 healthy controls | 2008/2009 or 2009/2010 influenza vaccine (CSL Biotherapies) 2009 pandemic influenza A (H1N1) vaccine  Subset: PPV (Pneumovax II®; Sanofi Pasteur) | NI | FC, ELISA | w4, m2, m3, ELISA. | moderate |
| Kersun et al. | 2013 | 110 ALL patients on chemotherapy 67 solid tumor patients on chemotherapy | Seasonal inactivated trivalent influenza vaccine (2006-2007, 2007-2008, 2008-2009 or 2009-2010) (manufacturer not specified) | NI | FC, HAI assay | m2, m4, y1. HAI assay. | moderate |
| Reilly et al. | 2013 | 8 AML patients on chemotherapy NI healthy controls | Trivalent influenza vaccine (manufacturer not specified) | NI | FC, HAI assay, ELISpot | m2, m4 and y1. HAI assay, ELISpot. | moderate |
| Koskenvuo et al. | 2016 | 9 ALL patients on NOPHO chemotherapy | PCV (Prevenar 7®; Wyeth) | m6-12 post-treatment | FC | NI | moderate |
| Goswami et al. | 2017 | 10 AML patients on heterogeneous chemotherapy | TIV 2012–2013 (manufacturer not specified) | NI | FC, MN assay, BCR spectratyping | d30. MN assay, ELISpot. | moderate |
| Struijk et al. | 2010 | Post-kidney Tx patients on prednisolone 12 on cyclosporine 12 on MPA 12 on everolimus  13 healthy controls | Immucothel® (biosyn) PPV (Pneumovax®; N/R) Tetanus toxoid (Sanofi Pasteur) | NI | FC, ELISA | d14, ELISA. | low |
| Cowan et al. | 2014 | 22 post-kidney Tx patients on immunosuppression 21 healthy controls | Fluvirin® (Novartis) or Fluzone® (Sanofi Pasteur) | NI | FC, ELISpot, ELISA | d7, d14, d28. ELISA, ELISpot, FC. | moderate |
| Egli et al. | 2015 | 47 post-organ Tx patients on heterogeneous immunosuppression 11 healthy controls | Intanza® (Sanofi Pasteur) or Vaxigrip® (Sanofi Pasteur) | NI | FC, HI assay | w4. HAI assay. | moderate |
| Kobie et al. | 2011 | RA, patients 61 on anti-TNF  70 on MTX 33 untreated 97 healthy controls | Seasonal inactivated trivalent influenza vaccine (manufacturer not specified) | NI | FC, HAI assay, ELISpot | d5-7, d8-10, m1 and m6, HAI assay, ELISpot, FC. | moderate |
| Kamphuis et al. | 2013 | 32 sarcoidosis patients on heterogeneous immunosuppression  28 healthy controls | Agriflu® (Novartis) Focetria® (Novartis) PPV (Pneumo 23®; N/R) Hib vaccine (not specified) | NI | FC, HAI assay, nephelometry | w4, HAI assay, NI for evaluation of other vaccines. | moderate |
| Salinas et al. | 2013 | 41 SpA patients on TNF-blocking immunosuppression  15 untreated SpA patients | Engerix-B® (GSK) + revaccination at W6 and W22 PPV (Pneumovax 23®; Merck) | NI | FC, IgκRHEMA assay, ELISA | w6, w10, w22, w26. ELISA. | low |
| Fallahi et al. | 2014 | 18 IBD patients on heterogeneous immunosuppression 20 healthy controls | PPV (Pneumo 23®; Sanofi Pasteur) | NI | FC, ELISA | d28, ELISA. | moderate |
| Heijstek et al. | 2014 | 63 JIA patients on heterogeneous immunosuppression  49 healthy controls | Cervarix® (GSK) | m0, m1, m6 | ELISpot, multiplex assay | m3, m7, m12. Multiplex assay, ELISpot. | moderate |
| Bingham et al. | 2015 | RA patients on MTX  51 on tabalumab 17 on placebo | Boostrix® (GSK)  PPV (Pneumovax 23®; Merck) | w24 after drug start | FC, ELISA | w4, ELISA.  w28, FC. | some concerns |

Abbreviations: NI , not indicated; alloHCT, allogeneic hematopoietic stem cell transplantation; IVIG, intravenous immunoglobulins; NHL, non-Hodgkin lymphoma; CP -CML, chronic phase - chronic myeloid leukemia; TKI, tyrosine kinase inhibitor; ITP, immune thrombocytic purpura; SCID, severe combined immunodeficiency; ALL, acute lymphoblastic leukemia; AML, acute myeloid leukemia; SpA, spondylarthritis; Tx, transplantation; JIA, juvenile idiopathic arthritis; RA, rheumatoid arthritis; MTX, methotrexate; IBD, inflammatory bowel disease; CP, cyclosporine; MPA, mycophenolate sodium; TT, tetanus toxoid; TIV, trivalent influenza vaccine; PPV, pneumococcal polysaccharide vaccine; PCV, pneumococcal conjugate vaccine; Hib, *H. influenzae* type b; FC, flow cytometry; ELISA, enzyme-linked immunosorbent assay; ELISpot, enzyme-linked immune absorbent spot; HAI, hemagglutination inhibition; OPK, opsonophagocytic killing; d, day; w, week; m, month; y, year; BCR, B-cell receptor; DC, dendritic cell.
